# Early Neutrophilia Marked by Aerobic Glycolysis Sustains Host Metabolism and Delays Cancer Cachexia

**DOI:** 10.3390/cancers14040963

**Published:** 2022-02-15

**Authors:** Michele Petruzzelli, Miriam Ferrer, Martijn J. Schuijs, Sam O. Kleeman, Nicholas Mourikis, Zoe Hall, David Perera, Shwethaa Raghunathan, Michele Vacca, Edoardo Gaude, Michael J. Lukey, Duncan I. Jodrell, Christian Frezza, Erwin F. Wagner, Ashok R. Venkitaraman, Timotheus Y. F. Halim, Tobias Janowitz

**Affiliations:** 1 Medical Research Council Cancer Unit, University of Cambridge, Hutchison/MRC Research Centre, Cambridge Biomedical Campus, Cambridge CB2 0XZ, UK; mp753@cam.ac.uk (M.P.); mf635@cam.ac.uk (M.F.); dp432@cam.ac.uk (D.P.); edoardogaude@gmail.com (E.G.); cf366@cam.ac.uk (C.F.); 2 Cold Spring Harbor Laboratory, Cold Spring Harbor, NY 11724, USA; skleeman@cshl.edu (S.O.K.); mouriki@cshl.edu (N.M.); lukey@cshl.edu (M.J.L.); 3 CRUK Cambridge Institute, University of Cambridge Li Ka Shing Centre, Cambridge CB2 0RE, UK; Martijn.Schuijs@cruk.cam.ac.uk (M.J.S.); shwethaa.raghunathan@cruk.cam.ac.uk (S.R.); Duncan.Jodrell@cruk.cam.ac.uk (D.I.J.); Tim.Halim@cruk.cam.ac.uk (T.Y.F.H.); 4 Biomolecular Medicine, Division of Systems Medicine, Department of Metabolism, Digestion and Reproduction, Imperial College London, London SW7 2AZ, UK; zoe.hall@imperial.ac.uk; 5 Metabolic Research Laboratories, Wellcome Trust-MRC Institute of Metabolic Science, Cambridge Biomedical Campus, Cambridge CB2 0QQ, UK; mv400@medschl.cam.ac.uk; 6 Laboratory Genes and Disease, Department of Laboratory Medicine Department of Dermatology, Medical University of Vienna (MUV), 1090 Vienna, Austria; erwin.wagner@meduniwien.ac.at; 7 Laboratory Genes and Disease, Department of Dermatology, Medical University of Vienna (MUV), 1090 Vienna, Austria; 8 Cancer Science Institute of Singapore, National University of Singapore, Singapore 117599, Singapore; 9 Agency for Science, Technology and Research (A*STAR), Singapore 138648, Singapore; 10 Northwell Health Cancer Institute, New Hyde Park, NY 11042, USA

**Keywords:** cancer, cachexia, metabolism, host, neutrophils, aerobic glycolysis

## Abstract

**Simple Summary:**

Patients with cancer suffer from systemic metabolic impairment during cancer progression. An elevated neutrophil–lymphocyte ratio is a negative predictor of outcome. In the present study, we investigate a potential role of neutrophils on host metabolism. We identified widespread neutrophilia as an early event in cancer progression and found that neutrophils display an enhanced aerobic glycolytic profile. Pharmacological inhibition of aerobic glycolysis, a pathway that also characterizes cancer cells, leads to expanded neutrophilia, reduced tumor size, and shorter survival. Quantitative depletion of neutrophils impairs glucose homeostasis and the availability of hepatic lipids. Our results suggest that neutrophils play an adaptive role in metabolic host homeostasis during cancer progression and demonstrate that assessment of candidate cancer treatment efficacy should include both tumor and host responses.

**Abstract:**

An elevated neutrophil–lymphocyte ratio negatively predicts the outcome of patients with cancer and is associated with cachexia, the terminal wasting syndrome. Here, using murine model systems of colorectal and pancreatic cancer we show that neutrophilia in the circulation and multiple organs, accompanied by extramedullary hematopoiesis, is an early event during cancer progression. Transcriptomic and metabolic assessment reveals that neutrophils in tumor-bearing animals utilize aerobic glycolysis, similar to cancer cells. Although pharmacological inhibition of aerobic glycolysis slows down tumor growth in C26 tumor-bearing mice, it precipitates cachexia, thereby shortening the overall survival. This negative effect may be explained by our observation that acute depletion of neutrophils in pre-cachectic mice impairs systemic glucose homeostasis secondary to altered hepatic lipid processing. Thus, changes in neutrophil number, distribution, and metabolism play an adaptive role in host metabolic homeostasis during cancer progression. Our findings provide insight into early events during cancer progression to cachexia, with implications for therapy.

## 1. Introduction

Clinical studies have identified the consequences of inflammation, such as changes in the neutrophil–lymphocyte ratio (NLR) as an early biomarker of cancer progression [1,2]. Sustained systemic inflammation persists throughout cancer progression and mediates many tumor-host interactions. The cytokines and chemokines of the inflammatory responses in tumors not only contribute to the organization of the tumor microenvironment [3,4,5,6], but also systemically regulate non-tumoral host tissues and their metabolism [7,8]. In particular, systemic inflammation has been implicated in cancer cachexia, which is a common late manifestation of cancer progression that is defined by wasting of lean body mass and significant weight loss and is associated with poor prognosis [9]. In patients with cancer and in murine models, elevated interleukin-6 (IL-6) has been causally linked to cachexia and altered macronutrient processing. Mechanistically, IL-6 induces ketogenic impairment due to reprogramming of the liver [7] and browning of adipose tissue [8]. Also, IL-6 impairs intestinal barrier function in mouse models of cachexia, leading to translocation of microbial compounds [10], which can promote systemic inflammation [11]. Evidence of this reprogramming is already detectable in the pre-cachectic stage. Despite these clear implications of inflammation and the resulting immunological changes in the host, a potential contribution of the immune system to the host biology during cancer progression and ultimately the onset of cancer cachexia has not been systematically investigated. Efforts to understand the cellular and systemic processes that precede and drive the development of cancer progression to cachexia, as well as the biology of the pre-cachectic state, are important as overt skeletal muscle catabolism marks an irreversible endpoint of the disease [12,13].

Here, we characterize the temporal sequence of quantitative and qualitative changes in neutrophils during cancer progression and their role in the pathophysiology of cachexia. Using in vivo models that recapitulate the progression of cancer in humans, we report an early and robust increase in the circulating neutrophils and widespread neutrophilia in many solid organs that precedes end-stage cachexia. These neutrophils display an enhanced aerobic glycolytic profile that both cancer and immune cells are metabolically dependent on [14,15], highlighting the need to identify systemic consequences when targeting this pathway therapeutically. The pharmacological inhibition of aerobic glycolysis resulted in reduced tumor growth but shortened survival due to early onset cachexia. Targeted depletion of neutrophils revealed a role of inflammatory neutrophilia in lipolysis, lipid accumulation in the liver, and systemic glucose homeostasis. Taken together, our results position neutrophil metabolism at the interface of the metabolic interactions between cancer and host.

## 2. Materials and Methods

### 2.1. Laboratory Animals

There were two different mouse models of cachexia that were used: a transplanted C26 model of colorectal cancer and the genetically engineered autochthonous KPC model of pancreatic cancer. The C26 model is based on wild-type BALB/c mice that are inoculated subcutaneously with a syngeneic tumor. In the KPC system, an activating point mutation (G12D) in Kras and a dominant negative mutation in Trp53 (R172H) are conditionally-activated in the pancreas by means of Cre-Lox technology. Both preclinical models have been shown to develop tumors that secrete IL-6, therefore, the host is unable to produce ketones during the caloric deficiency that is associated with cachexia, causing a rise in glucocorticoid levels.

KPC and BALB/c mice were obtained from Charles River Laboratories. All the mice were kept in pathogen-free conditions on a 24 h 12:12 light-dark cycle and allowed to acclimatize for 7 days. Their body weight, food intake, and clinical signs were monitored on a daily basis. Handling was kept to a minimum. The mice were defined as cachectic and were sacrificed when showing clinical signs of sickness or when their weight loss exceeded 10% peak weight (usually corresponding to the starting body weight at day 0). Death was confirmed by cervical dislocation. The experiments and care of C26 syngeneic colon cancer mice and control mice, and KPC and PC were performed in accordance with national and institutional guidelines and approved by the UK Home Office, the animal ethics committee of the University of Cambridge.

#### 2.1.1. In Vivo Models

Tumor development in KPC mice was monitored and detected via high-resolution ultrasound scans (Vevo 2100, VisualSonics, Toronto, ON, Canada). Weight-stable, PDAC-bearing KPC mice with tumors 3–5 mm in size and no evidence of obstructive common bowel duct were enrolled in the experiments together with their weight- and age-matched PC controls. Weight-stable wild-type male BALB/c mice were inoculated subcutaneously in their right flank with 2 × 10^6^ viable C26 colorectal cancer cells in RPMI vehicle at 100 μL per mouse. The C26-injected BALB/c mice were enrolled in the study together with their respective weight- and age-matched non-tumor-bearing BALB/c control littermates.

#### 2.1.2. Heptelidic Acid Treatment

For pharmacological inhibition of aerobic glycolysis, C26 syngeneic colon cancer mice and control mice received daily intraperitoneal injections with heptelidic acid (1 mg/kg; Cayman Chemical, Ann Arbor, MI, USA), starting on day 14 post-subcutaneous injection of C26 cells.

Toxicity testing of heptelidic acid in the C26 and control mice was performed on day 4 after the start of treatment. Cardiac blood punctures were collected, allowed to clot at room temperature (15–30 min), and then the clot was removed by centrifuging at 1000–2000× *g* for 10 min in a refrigerated centrifuge (4 °C). The resulting supernatant sample was designated serum and was used for the toxicity tests that were performed by IDEXX Laboratories, Inc., Westbrook, ME, USA.

GAPDH activity measurements in the liver, kidney, and pancreas was performed on day 4 after the start of treatment with heptelidic acid in the C26 and control mice. Tissues were collected and kept in ice cold PBS during dissection. Lysis and homogenization of 10 mg of each fresh tissue in GAPDH Assay Buffer was performed using 5 mm Stainless Steel Beads and TissueLyser II (Qiagen, Hilden, Germany). The GAPDH activity was measured using a GAPDH Activity Assay Kit (#K680-100, BioVision, Milpitas, CA, USA).

#### 2.1.3. Neutrophil Depletion

The C26 tumor-bearing mice were injected intraperitoneally with 200 µg of GR1-depleting antibody (Ultra-LEAF anti-mouse-Ly6G/Ly6C Ab, BioLegend, San Diego, CA, USA). The control mice were injected intraperitoneally with 200 µg of isotype control (Ultra-LEAF rat IgG2b, BioLegend). The mice were injected once with GR1-depleting antibody or isotype control on day 12 post- C26 cancer cell subcutaneous injection.

#### 2.1.4. Total Food Restriction (TFR)

The mice were singly-housed and moved into new cages containing only bedding, nesting, material, and a water bottle. TFR was initiated in the middle of the 12-h light period at 12:00 p.m., and the mice were re-fed after 24 h.

#### 2.1.5. Tumor Size

PDAC tumors were detected via palpation and confirmed by high-resolution ultrasound imaging (Vevo 2100, VisualSonics, Toronto, ON, Canada), and ultimately at necropsy. Tumor growth was monitored by ultrasound scans that were assessed at multiple angles. The mice were carefully observed for any macroscopic metastases. Tumor development in BALB/c mice was determined via palpation and monitored daily by caliper measurements. The maximum cross-sectional area and maximum diameter of the tumors were determined for each time point.

#### 2.1.6. Plasma Samples

Tail bleeds and terminal cardiac bleeds were taken. The tail vein bleeds were performed using a scalpel via tail venesection without restraint, and the terminal bleeds were obtained through exsanguination via cardiac puncture under isoflurane anesthesia. Samples were kept on ice at all times. Plasma samples were collected into heparin-coated capillary tubes to avoid coagulation and were processed as follows: centrifuge spin at 14,000 rpm for 5 min at 4 °C, snap frozen in liquid nitrogen, and stored at −80 °C. Analyses of alanine aminotransferase (ALT), aspartate aminotransferase (AST), triglycerides, free fatty acids (FFA), glucose, and insulin were performed by the Core Biochemical Assay Laboratory, University of Cambridge.

#### 2.1.7. Tissue Collection

PDAC tumors, liver, spleen, quadricep muscle, subcutaneous fat, and lungs were collected and weighed during necropsy dissection. PDAC tumors, liver, spleen, quadricep muscle, subcutaneous fat, lungs, and hypothalamus were collected and weighed during necropsy dissection. Subsequently, the tumor, liver, and spleen samples were cut into two equal parts, which were either snap frozen in liquid nitrogen or fixed in 10% neutral buffered formaldehyde for 24 h at room temperature before being transferred to 70% ethanol and later paraffin-embedded or immunohistochemistry processing. All the other organs and tissue samples were immediately snap frozen and stored at −80 °C.

### 2.2. RNA Isolation, Reverse Transcription, and Quantitative RT-PCR (qRT-PCR)

The frozen tissue samples were lysed in 1 mL of TRIzol (Sigma, Burlington, MA, USA) using a Precellys^®^ tissue homogenizer. A total of 200 μL of chloroform was added, and the samples were centrifuged for 15 min at 13,200 rpm at 4 °C. The aqueous phases containing the RNA were carefully transferred to new tubes, and 500 μL of isopropanol were added for precipitation of the RNA. After vigorously shaking, the samples were centrifuged for 30 min at 13,200 rpm at 4 °C. The supernatants were removed, and the samples were washed by adding 1000 μL of cold 80% ethanol followed by centrifugation at 13,200 rpm for 10 min at 4 °C. Once dried, the pellets were dissolved in nuclease-free water; the added volume depended on the pellet size. Then, the samples were stored at −80 °C until they were used. To purify the RNA samples, DNAse treatment was performed: 1–2 μg of RNA were diluted in a total volume of 10 μL, including 1 μL of reaction buffer and 1 μL of DNAse I (1U/μL), and nuclease-free water. After incubating for 15 min at room temperature, 1 μL of 25 mM EDTA was added to inactivate the reaction. A total of 10 μL of GoScriptTM Reverse Transcription Mix was prepared for each cDNA reaction (4 μL of nuclease-free water, 4 μL of GoScriptTM Reaction Buffer, Oligo (dT), and 2 μL of GoScriptTM Enzyme Mix (Promega, Manufacturer, Madison, Wis., USA). This mastermix was combined with the RNA sample (final volume of 20 μL) and, after mixing well, the reaction was incubated following these steps: (1) anneal primer (5 min at 25 °C), (2) extension (60 min at 42 °C), and (3) inactivation (15 min at 70 °C). The samples were stored at −20 °C until qPCR was performed. The Quantitative PCR Primer Database (QPPD) was used to search for primers that were previously validated in the literature (Available online: https://pga.mgh.harvard.edu/primerbank/ (accessed on 29 October 2021). The NCBI Standard Nucleotide BLAST online tool was used to verify the primers (Available online: https://blast.ncbi.nlm.nih.gov/Blast.cgi?PAGE_TYPE=BlastSearch (accessed on 29 October 2021). A total of 2 μL of cDNA, 5 μL of Sybr Green qPCR Master mix, 3 μL of nuclease-free water, and 0.2 μL of primers (0.1 μM) were used per reaction. The comparative cycle threshold method was used for quantification, and expression levels were normalized using the housekeeping gene β-actin. All the samples were run in technical triplicate. The primer sequences are available upon request.

### 2.3. Single Cell Preparation

The cell suspensions were prepared from tissues by mechanical dissociation, followed by digestion in 5 mL of RPMI-1640 containing collagenase I (500 U/mL) and DNase I (0.2 mg/mL) for 45 min at 37 °C on a shaker (220 rpm), followed by filtration through a 70-μm strainer and 25% Percoll gradient enrichment of leukocytes, and red blood cell (RBC) lysis. The tumor cells were recovered without Percoll enrichment. The blood cells were lysed in 5 mL of RBC lysis buffer three times for 5 min, and the spleens were strained through a 70-μm filter in RPMI-1640 before lysing erythrocytes with RBC lysis buffer for 5 min. Single cells were restimulated and stained for surface and intracellular markers (see flow cytometry).

### 2.4. Flow Cytometry

Single cells were incubated with anti-mouse CD16/32 (Thermo Fisher, Waltham, MA, USA) to block the Fc receptors and stained as indicated. The lineage cocktail contained ±CD3 (145-2C11), ±NK1.1 (PK136), TCRβ, CD5 (53-7.3), CD19 (1D3), CD11b (M1/70), CD11c (N418), FcεR1α (MAR-1), F4/80 (BM8), Ly-6C/G (Rb6-8C5), and Ter119 (TER-119) all on eFluor450 (eBioscience, San Diego, CA, USA). For intracellular staining, we used the Foxp3/Transcription Factor Kit (Thermo Fisher) or Cytofix/Cytoperm Kit (BD Biosciences, Franklin Lakes, NJ, USA) as per the manufacturers’ instructions. For intracellular cytokine detection, single cells were stimulated with PMA (60 ng/mL) and ionomycin (500 ng/mL) plus 1 × protein transport inhibitor (Thermo Fisher), 1× cytokine stimulation cocktail (Thermo Fisher), or plate-bound anti-NK1.1 mAb (10–30 µg/mL, BioXcell, Lebanon, NH, USA), or recombinant IL-12 (20 ng/mL) and IL-18 (5 ng/mL) in culture media (RPMI-1640, 10% FCS) at 37 °C for 3 h before staining. The data were acquired on a BD Fortessa or Symphony instrument (BD Biosciences, City, State, Country), and cells were quantified using CountBright beads. Data were analyzed using FlowJo X (Tree Star, v10, Ashland, OR, USA). B220 (RA3-6B2, Life Technologies, APC.eFl780), CD3e (145-2C11, eBioscience, PE.Cy7 and eFl450) (25-0031-83, 4304567), CD4 (RM4-5, eBioscience, AF700), CD5 (53-7.3, eBioscience, eFl450), CD8 (53-6.7, eBioscience, PerCP.eFl710 and SB645), CD11b (M1/70, eBioscience, eFl450, APC.eFl780, and BV785), CD11c (N418, eBioscience, eFl450 and AF700), CD16/32 (93, BioLegend), CD19 (1D3, eBioscience, eFl450), CD31 (390, BioLegend, BV605), CD45 (30-F11, BioLegend, BV510), CD64 (X54-5/7.1 BioLegend, San Diego, CA, USA BV711), CD127 (SB/199, BD Biosciences, PE.CF594), CD172a (P84, BioLegend, AF488), c-Kit (BioLegend), EpCam (G8.8, BioLegend, BV711), FceR1a (MAR-1, eBioscience, eFl450 and PerCP.eFl710), Fixable Viability Dye (eBioscience, UV455), F4/80 (BM8, eBioscience, eFl450 and APC.eFl780), I-A/I-E (CI2G9, BD Biosciences, BUV395), Ly-6C/G (Rb6-8C5, eBioscience, eFl450), Ly-6G (1A8-Ly6g, eBioscience, PE.eFl610), Ly-6C (HK1.4, eBioscience, PE.Cy7), NK1.1 (PK136, BD Biosciences, BUV395 and eBioscience, eFl450), RELMα (DS8RELM, Invitrogen, Waltham, MA, USA), Podoplanin (8.1.1, BioLegend, PE.Cy7), Roryt (Q31.378, eBioscience, PerCP.eFl710), SiglecF (1RNM44N, eBioscience, SB600), and Ter119 (TER-119, eBioscience, eFl450).

### 2.5. Histology

The lung and liver lobes were fixed in 10% neutral-buffered formalin in phosphate-buffered saline (PBS) for 24 h, followed by transfer to 70% ethanol in PBS for another 24 h and embedded into paraffin. Then, 3-μm sections were cut and stained with anti-myeloperoxidase antibody (Abcam, ab9535, Cambridge, UK). The CRUK-CI Histology Core performed tissue embedding, sectioning, and staining. Image quantification was performed using the HALO software (HALO, Indica labs, Albuquerque, NM, USA). Oil Red O and Periodic Acid-Schiff’s (PAS) stainings were performed by the Metabolic Research Laboratories, University of Cambridge. For the Oil Red O staining, fresh frozen sections were fixed in 10% neutral-buffered formalin for 10 min, then stained in Stock Oil Red O plus Dextrin solution for 20 min and counterstained with Mayer hematoxylin for 30 s. For the PAS staining, 2–5 µm sections were incubated with Periodic Acid Solution for 5 min, then Schiff’s reagent for 15 min, and counterstained with hematoxylin for 15 s.

### 2.6. Lipidomics

Liver tissue was homogenized in chloroform/methanol (2:1, 1 mL) using TissueLyser (Qiagen Ltd., Manchester, UK). Deionized water (400 uL) was added and, after mixing, a centrifugation step (13,000× *g*) was used to separate the organic lipid-containing layer. This was analyzed by liquid chromatography mass spectrometry (LC-MS) on an Accela Autosampler that was coupled to an LTQ Orbitrap EliteTM (Thermo Fisher Scientific, Hemel Hempstead, UK). Separation was achieved using an Acquity C18 BEH column at 55 °C. Mobile phase A was 60:40 acetonitrile:water, and mobile phase B was 90:10 isopropanol:acetonitrile, each with 10 mM ammonium formate. A gradient flow at 0.5 mL/min was used, starting with 40% B, to 99% B over 8 min, then held at 99% B for 0.5 min, then returned to the initial conditions. The HESI source conditions were 375 °C and 380 °C for the source and capillary temperature, respectively; the gas flow was 40 arbitrary units. Analysis was performed in positive ion mode by full scan (range *m*/*z* 200–2000). Triacylglycerol (TAG) identification was performed by accurate mass and retention time using an in-house database. The peak areas were normalized to an isotopically-labelled internal standard and tissue weight and summed for total TAG quantification.

### 2.7. MitoTracker

The neutrophils were isolated with a Neutrophil Isolation Kit (Miltenyi Biotec, Bergisch Gladbach, Germany) according to the manufacturer’s instructions. MitoTracker analysis was performed according to the manufacturer’s instructions (Deep Red FM molecular probes, Invitrogen, Waltham, MA, USA). For immunofluorescence, Anti-CPT1 antibody [8F6AE9] (Alexa Fluor^®^ 488, Abcam, ab171449, Cambridge, UK) was used. Immunofluorescence images were analyzed using Fiji software. The images were binarized, and a mask was created to segment the cells in 2D. The segmented cells were saved as regions of interest and were used to measure the mean intensity value of MitoTracker and CTP1. For each neutrophil, a unique averaged fluorescence intensity value was generated.

### 2.8. RNA Sequencing Data and Analysis

Bulk RNA sequencing data (FASTQ files) corresponding to neutrophils that were isolated from the spleen of wild-type C57BL/6J and KPC mice was downloaded from the Sequence Read Archive (SRA) (accession PRJNA749728) [16]. The reads were aligned using STAR to the GRCm39 mouse reference gene with GENCODE M27 annotations using the nf-core/rnaseq analysis pipeline version 3.0 [17]. Gene-level expression quantification was performed using RSEM. The count data were imported into edgeR and TMM-normalized prior to differential expression analysis between the wild-type and KPC neutrophils using the ‘glmQLFTest’ function in edgeR. Gene set enrichment analysis (GSEA) was implemented using the fgsea package for R, using the MSigDB Hallmark gene sets that were mapped to mouse orthologs that were available online: http://bioinf.wehi.edu.au/software/MSigDB/ (accessed on 29 October 2021).

### 2.9. Oxygen Consumption (OCR) and Extracellular Acidification Rates (ECAR)

To assess the oxygen consumption rate (OCR) and extracellular acidification rate (ECAR), each well of a XFe24 (Agilent, Santa Clara, CA, USA) cell culture microplate was coated with 25 µL of a solution containing 22.4 µg/mL CellTak (Corning, NY, USA). The solution was allowed to react for 20 min at room temperature and was either used immediately for the experiments or stored at 4 °C. A coated microplate was pre-warmed at 37 °C and 3 × 10^5^ cells were seeded in 675 µL of bicarbonate-free Dulbecco’s Modified Eagle’s Medium (DMEM) (D5030, Sigma-Aldrich, St. Louis, MO, USA) that was supplemented with 1 mM pyruvate, 4 mM glutamine, 40 mM phenol red, and 1% *v*/*v* fetal bovine serum (FBS). To eliminate carbonic acid residues from the medium, the cells were incubated for at least 30 min at 37 °C with atmospheric CO_2_ in a non-humidified incubator. OCR and ECAR were assayed in a Seahorse XF-24 extracellular flux analyzer by the addition via ports A–D of 25 mM Glucose (port A), 1 mM oligomycin (port B), 1 mM carbonyl cyanide-p-trifluor-omethoxyphenylhydrazone (FCCP, port C), 1 mM rotenone, and 1 mM antimycin A (port D) in the same cell culture medium that was used for cell seeding. A total of three measurement cycles of 2 min mix, 2 min wait, and 4 min measure were carried out at basal condition and after each injection.

### 2.10. Statistical Analysis

The data are expressed as the mean ± SEM unless otherwise stated and statistical significance was analyzed using GraphPad Prism software, (7.03 version, GraphPad, San Diego, CA, USA). For survival analysis, data were shown as Kaplan–Meier curves, and the log-rank (Mantel–Cox) test was used to assess survival differences. For quantitative data (organ weights), an unpaired two-tailed Student’s *t*-test was applied with Welch’s correction (does not assume equal standard deviations; SDs). When comparing more than 2 groups at the same time, a one-way ANOVA with Tukey’s correction for post hoc testing was used. For statistical comparison of quantitative data at different times (body weight), unpaired two-tailed Student’s *t*-tests were performed at each time point with the Holm–Šidák method correction for multiple comparisons.

## 3. Results

### 3.1. Widespread Neutrophilia Is an Early Systemic Manifestation of Cancer Progression

**Figure 1:**
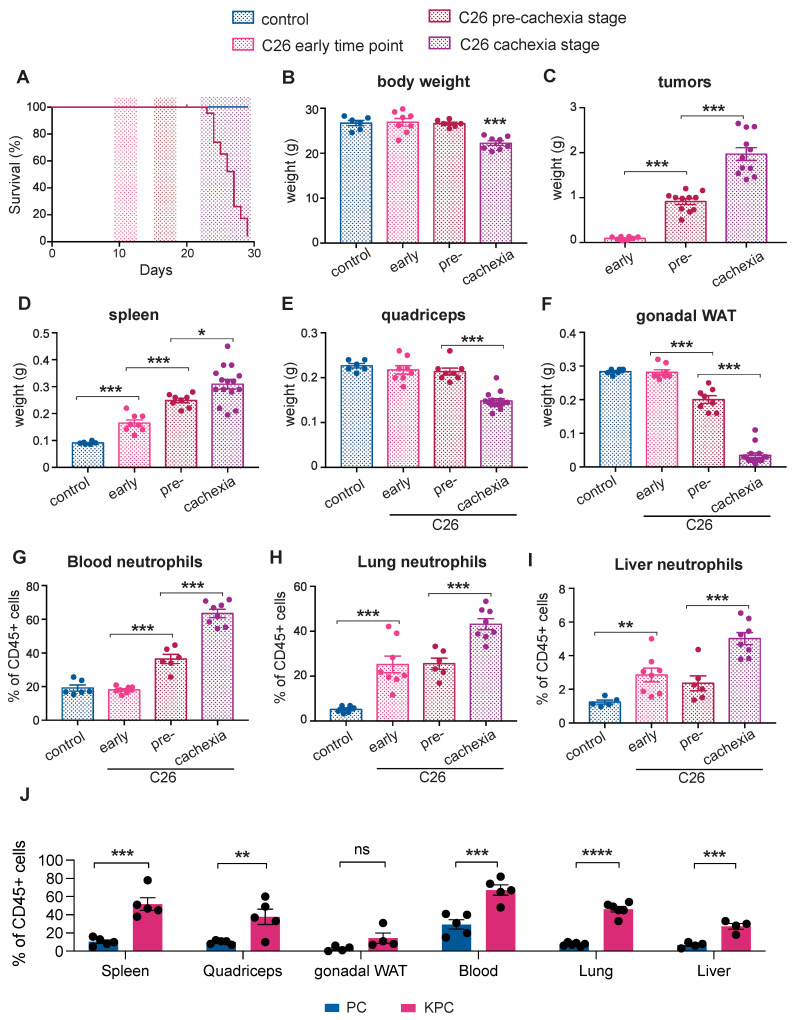
Immune changes during cancer progression in mouse models of cachexia. (**A**) The mice were injected with C26 colorectal cancer cells and sacrificed at three distinct time points: an early time point (9–10 days post-tumor inoculation), the pre-cachectic stage (15–16 days post-injection), and when cachexia occurred (≥21 days post-injection); (**B**–**F**) The body (**B**), tumor (**C**), spleen (**D**), quadriceps (**E**), and gonadal white adipose tissue (gWAT) (**F**) weights of mice that were sacrificed at the time points that are defined in Figure 1A; (**G**–**I**) Quantification of neutrophils (displayed as % of neutrophils out of all CD45+ cells), by flow cytometry in the blood (**G**), lung (**H**), and liver (**I**) of mice that were sacrificed at the time points that are defined in Figure 1A; (**J**) Quantification of neutrophils (displayed as % of neutrophils out of all CD45+ cells) in the spleen, quadriceps, gWAT, blood, lung, and liver of pre-cachectic KPC mice and the PC controls. The data are expressed as the mean ± SEM. A one-way ANOVA with Tukey’s correction for post hoc testing was used in (**B**–**I**). Statistical differences in (**J**) were examined using unpaired two-tailed Student’s *t*-test with Welch’s correction. * *p*-value < 0.05, ** *p*-value < 0.01, *** *p*-value < 0.001, **** *p*-value < 0.0001, ns: not significant.

An increase in the NLR is a well-recognized prognostic marker in patients with advanced cancer [18] and is associated with cachexia [19]. We utilized the non-metastatic subcutaneous colorectal cancer-derived C26 model, a well-validated murine model of epithelial cancer-induced cachexia that is associated with inflammation, to dynamically characterize alterations in neutrophils at different stages of the disease. The mice were sacrificed at three distinct time points of cancer progression (Figure 1A): an early time point (9–10 days post-injection of C26 cancer cells, the when mice had small tumors), the pre-cachectic stage (15–16 days post-injection, when the mice were weight stable with normal food intake), and during cachexia (≥21 days post-injection, when the mice displayed weight loss and reduced food intake) (Figure 1B,C and Figure S1A,B). Cachexia was defined by a > 10% decrease in the total body weight. Splenomegaly developed progressively from the early time point to the cachectic stage (Figure 1D). Cachectic mice exhibited a pronounced loss of gonadal white adipose tissue (gWAT) and muscle mass wasting, while pre-cachectic mice showed loss of gWAT but no muscle wasting; gonadal WAT and skeletal mass were unchanged at the early time point (Figure 1E,F). We subsequently performed detailed immunophenotyping of tumors, blood, and peripheral organs by flow cytometry to investigate the changes in immune profile that precede the onset of cachexia. We detected an increase of blood neutrophils starting at the pre-cachectic stage (Figure 1G). An analysis of tissue neutrophils showed that in the lung and liver of C26 tumor-bearing mice, the neutrophil numbers were already increased at the early time point (Figure 1H,I).

We also characterized systemic immune changes at the pre-cachectic stage in a genetically engineered, autochthonous murine model of pancreatic ductal adenocarcinoma (PDAC) that recapitulates human pathology, including the development of cachexia. The LSL-KrasG12D/+; LSL-Trp53R172H/+; Pdx1-Cre mutation-bearing (KPC) mice were sacrificed at a timepoint representing pre-cachexia, when tumors of 5–10 mm cross-sectional diameter were confirmed by ultrasound scanning (Figure S1C), but no body weight or muscle loss was evident when compared to the control age- and sex-matched Trp53R172H/+; Pdx1-Cre (PC) littermates (Figure S1D,E). Despite the absence of weight loss or sarcopenia, we observed a substantial systemic increase in neutrophils in all the analyzed tissues of pre-cachectic tumour-bearing mice compared to the non-tumour-bearing PC controls (Figure 1J). Other immune cell-types were not globally affected; however, eosinophils and macrophages were increased in tissues such as the lung, liver, and adipose tissue (Figure S6). Thus, neutrophilia was detectable in the blood and tissues of C26 mice during cancer progression and in pre-cachectic KPC mice compared to the PC controls (Figure 1G–J) resulting in an elevated NLR in the circulation (Figure S1F).

Consistent with the early migration of neutrophils into non-cancerous tissues, we detected increased myeloperoxidase (MPO), a neutrophil marker, in the hepatic and pulmonary parenchyma of pre-cachectic KPC mice, in areas where no microscopic metastatic growth was detectable (Figure S1G,I). Furthermore, gene expression analysis showed increased levels of neutrophil chemokines in the livers and lungs of the KPC mice, including Cxcl1, Cxcl2, and Cxcl5 (Figure S1H,J); a corresponding increased expression of Cxcr2 was observed in both organs, while Cxcr1 was marginally increased in the lung. In line with the flow cytometry data, we did not see an increase in the monocyte chemokine Ccl2. Liver transaminases were unchanged in the pre-cachectic KPC mice (Figure S1K,L), suggesting that neutrophil infiltration in the liver is not accompanied by signs of hepatocellular damage. No changes in the circulating lipids, glucose, or insulin levels were observed (Figure S1M–P).

Taken together, these findings show the dynamics and spatial distribution of immune changes that precede cachexia onset. Notably, quantifiable neutrophilia was detectable in established murine models of inflammation-associated epithelial cancer at an early time point during cancer progression, when the organism is otherwise seemingly unaffected by the tumor.

### 3.2. Pre-Cachectic Mice Exhibit Increased Bone Marrow and Splenic Extramedullary Granulopoiesis

**Figure 2:**
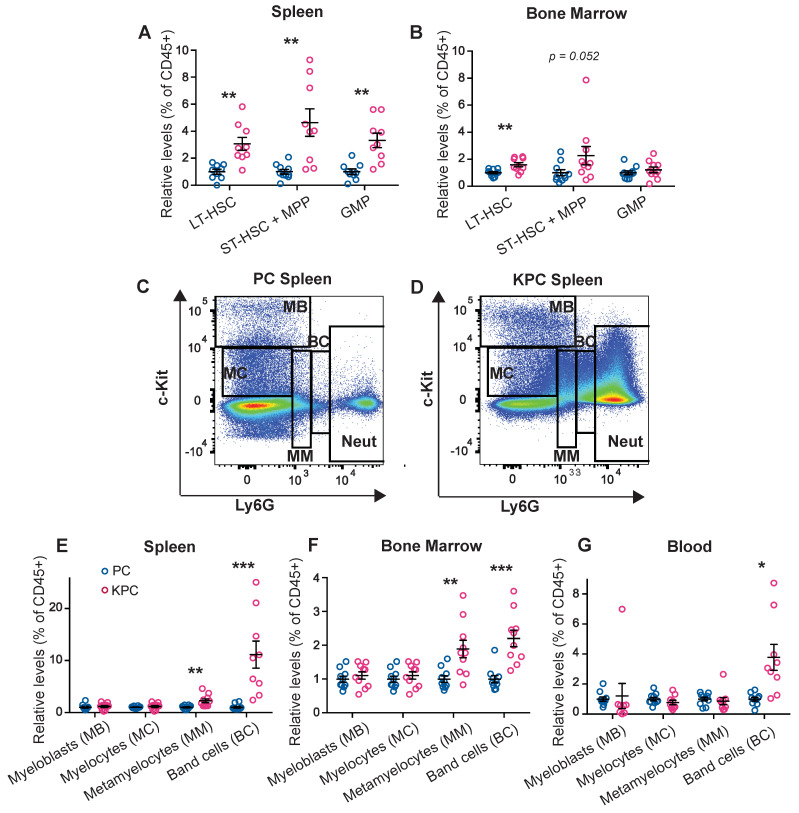
Quantification and FACS gating of progenitor cells, neutrophil precursors, and neutrophil populations in the KPC model. (**A**,**B**) Quantification of the HSC and progenitor cells (displayed as % out of all CD45+ cells) in the spleen (**A**) and bone marrow (**B**) of the pre-cachectic KPC and PC controls; (**C**,**D**) FACS gating strategy for neutrophils and neutrophil precursor populations in the spleen of the PC controls (**C**) and the pre-cachectic KPC mice (**D**); (**E**–**G**) Quantification of the neutrophil precursor populations (displayed as % out of all CD45+ cells) in the spleen €, bone marrow (**F**), and blood (**G**) of the pre-cachectic KPC mice and the PC controls. The data are expressed as the mean ± SEM. Statistical differences were examined using unpaired two-tailed Student’s *t*-test with Welch’s correction. * *p*-value < 0.05, ** *p*-value < 0.01, *** *p*-value < 0.001.

We next performed flow cytometry quantification to investigate the abundance of immune progenitor populations in the spleen and bone marrow, the main hematopoietic organs. The gating strategies that we used have been previously defined [20,21]. Compared to control PC mice, both the long-term (LT) and short-term (ST) hematopoietic stem cell (HSC) populations were increased in the spleen of pre-cachectic KPC mice, along with increased multipotent progenitor (MPP) and granulocyte-monocyte progenitor (GMP) populations; in contrast, only the LT-HSC population was increased in the bone marrow (Figure 2A,B). In the spleens of pre-cachectic KPC mice, we observed a significant increase in immature neutrophils/late progenitor cells, namely metamyelocytes and band cells (Figure 2C–E). Neutrophil late progenitor cell levels were also increased in the blood and bone marrow, although to a lesser extent (Figure 2F,G). The increased spleen size and abundance of splenic HSC and neutrophil progenitor cells suggest that splenic hematopoiesis is the primary origin of systemic neutrophilia in pre-cachectic KPC mice.

### 3.3. Neutrophils Display an Inflammatory Transcriptional Profile and Distinctive Aerobic Glycolytic Metabolism during Cancer Progression

**Figure 3:**
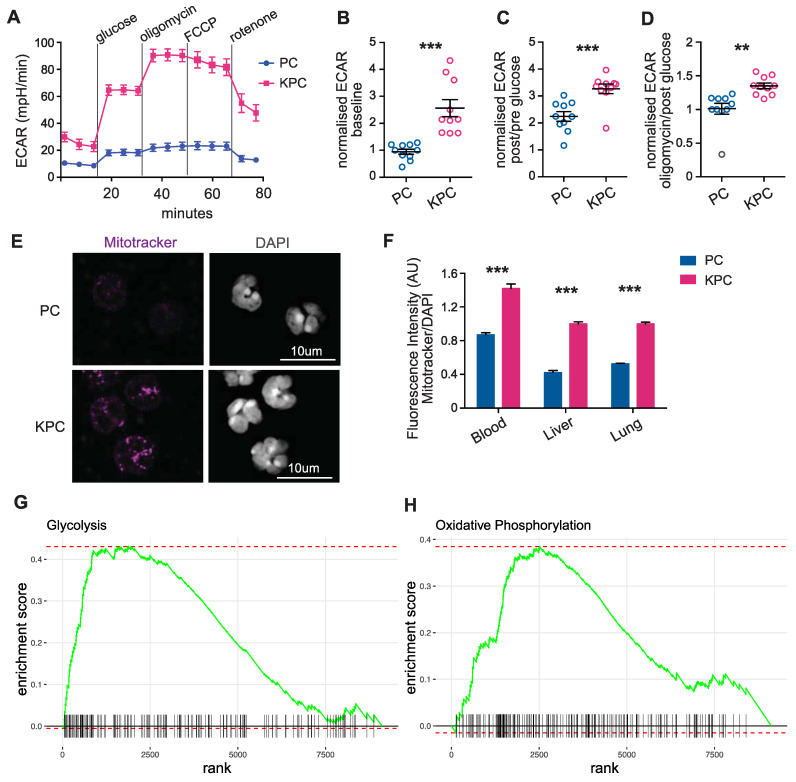
Characterization of immune cell metabolism in the pre-cachectic KPC mice. (**A**) Extracellular acidification rate (ECAR) of circulating leukocytes from pre-cachectic KPC mice and PC controls that were measured in real-time by Seahorse assay at baseline, after glucose administration, and after treatments with oligomycin, phenylhydrazone (FCCP), and rotenone; (**B**–**D**) Normalized ECAR measurements (ratio of compared timepoints) in the circulating leukocytes from mice in Figure 3D at baseline (**B**), after glucose administration (**C**), and oligomycin treatment (**D**); (**E**) Representative MitoTracker and DAPI immunofluorescence staining in the sorted circulating neutrophils from pre-cachectic KPC mice and PC controls; (**F**) Quantification of the fluorescence intensity (AU) of MitoTracker staining relative to DAPI staining in circulating, hepatic, and pulmonary neutrophils from mice in (**C**). (**G**,**H**) Gene set enrichment analysis (GSEA) for glycolysis (**G**) and oxidative phosphorylation (**H**)-related genes in isolated neutrophils of the KPC compared to the wild-type C57BL/6J mice. The data are expressed as the mean ± SEM. Statistical differences in (**B**–**D**,**F**) were examined using an unpaired two-tailed Student’s *t*-test with Welch’s correction. Statistical analysis in (**G**,**H**) is described in the Methods section. ** *p*-value < 0.01, *** *p*-value < 0.001.

We next analyzed the metabolic phenotype of the activated neutrophils in our model of pre-cachectic KPC mice. Neutrophils are, as first cellular responders of the innate immune system, very easily activated during sample processing, which can result in cell clumping and affect the neutrophil quality and quantity [22]. To preserve the metabolic profile of the cells, and as the leukocyte fraction was mostly comprised of neutrophils in the KPC mice, we used total leukocytes for cellular metabolic characterization. Glycolytic flux, that is measured by the extracellular acidification rate (ECAR), was significantly elevated in the circulating leukocytes from pre-cachectic KPC mice: at baseline; after glucose administration; and after independent treatments with the oxidative phosphorylation inhibitor oligomycin (a potent disruptor of ATP synthesis), with phenylhydrazone (FCCP) (interferes with proton gradient), and with rotenone (an inhibitor of the electron transport chain in mitochondria) (Figure 3A–D). The oxygen consumption rate (OCR) was also increased in the leukocytes from pre-cachectic KPC mice (Figure S2A,B). We then examined whether the circulating and tissue-infiltrating neutrophils were responsible for the observed changes. Neutrophils from the pre-cachectic KPC mice exhibited different mitochondrial activity than those from the control PC mice. Quantification of mitochondrial mass using MitoTracker (Deep Red FM) showed increased mitochondrial mass in the hepatic, pulmonary, and circulating neutrophils of the KPC mice (Figure 3E,F). In line with the enhanced mitochondrial metabolism that was observed in the leukocytes, isolated circulating neutrophils from the KPC mice showed increased staining for carnitine palmitoyltransferase 1 (CPT1), the enzyme that is responsible for transferring fatty acids inside the mitochondria (Figure S2C,D). Thus, neutrophils from pre-cachectic mice exhibited an increase in overall metabolism and reliance on glycolysis.

We aimed to confirm our findings by analyzing publicly available data on neutrophils, and, therefore, used bulk RNA sequencing data from splenic neutrophils that were isolated from wild-type C57BL/6J and KPC mice (SRA accession number PRJNA749728) [16]. Unsupervised differential gene expression analysis demonstrated the elevated expression of genes that are associated with hypoxia, apoptosis, fatty acid, and cholesterol metabolism, as well as the overexpression of genes that are related to glycolysis and oxidative phosphorylation in neutrophils of the KPC mice compared to neutrophils of the wild-type control mice without cancer (Figure S2E). Gene set enrichment analysis (GSEA) further identified increased expression in glycolysis and oxidative phosphorylation genes in the KPC-derived neutrophils relative to the wild-type (Figure 3G,H). These data support the hypothesis that neutrophils from pre-cachectic KPC mice undergo activation-induced metabolic changes.

The metabolic switch to aerobic glycolysis, termed the Warburg effect, has been observed in both activated immune cells and cancer cells. Here, we find that this transition characterizes neutrophils during cancer progression. Combined with the known utilization of Warburgian metabolism in cancer cells [15,23,24], these findings indicate that this metabolic state concurrently exists in both cell types within the same organism during cancer progression. Our results suggest that neutrophilia in cancer progression is an energy-expending process and may impact metabolic homeostasis systemically. To understand the relevance of aerobic glycolysis in the context of cachexia, we next targeted this pathway.

### 3.4. Targeted Inhibition of Aerobic Glycolysis Leads to Reduced Tumor Size and Expands Widespread Neutrophilia

Inhibiting aerobic glycolysis to down-modulate the immune response is a therapeutic strategy that is currently employed for psoriasis and multiple sclerosis [23]. Given the relevance of modified glucose metabolism to activated neutrophils and cancer cells, we assessed whether modulating aerobic glycolysis could affect cancer progression and the clinical severity of cancer-associated cachexia.

**Figure 4:**
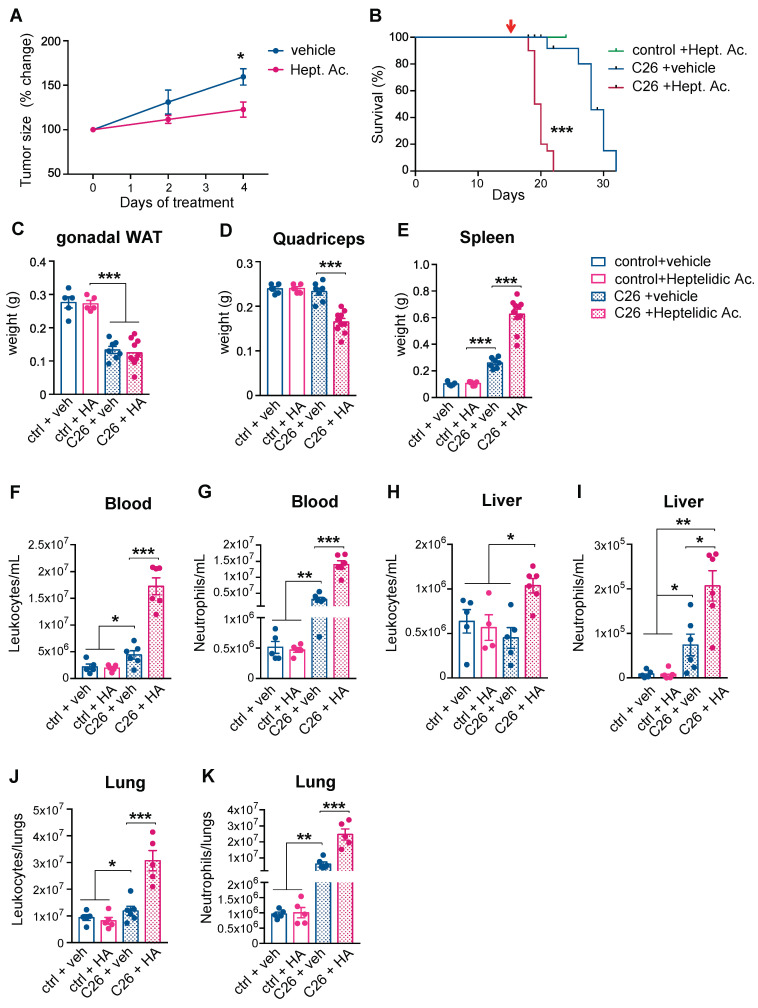
Effects of systemic inhibition of aerobic glycolysis on the severity of cancer cachexia. (**A**) Longitudinal tumor measurements of the C26 mice that were treated with heptelidic acid or the vehicle. The red arrow indicates start of treatment; (**B**) Overall survival of the C26 mice and littermates treated with heptelidic acid or vehicle; (**C**–**E**) The tissue weights of gonadal white adipose tissue (gWAT) (**C**), quadriceps (**D**), and spleen (**E**) of time-matched mice in (**A**); (**F**–**K**) Quantification of leukocyte (**F**,**H**,**J**) and neutrophil (**G**,**I**,**K**) counts in the circulation (**F**,**G**), the liver (**H**,**I**), and the lungs (**J**,**K**) of mice in (**A**). The data are expressed as the mean ± SEM. Unpaired two-tailed Student’s *t*-tests were performed at each time point in (**A**), with the Holm–Šidák method correction for multiple comparisons. Kaplan–Meier curves in (**B**) were statistically analyzed by using the log-rank (Mantel–Cox) test. A one-way ANOVA with Tukey’s correction for post hoc testing was used in (**C**–**K**). * *p*-value < 0.05, ** *p*-value < 0.01, *** *p*-value < 0.001.

Glyceraldehyde 3-phosphate dehydrogenase (GAPDH), the glycolytic enzyme that is rate-limiting exclusively in cells that rely on aerobic glycolysis, can be selectively inhibited by heptelidic acid [25,26,27,28]. Pre-cachectic C26 tumor-bearing mice and controls were treated daily with heptelidic acid or vehicle from day 14 post-subcutaneous injection of C26 cells. Treatment with heptelidic acid in the C26 pre-cachectic mice resulted in significantly reduced tumor growth rates relative to the C26 tumor-bearing mice that were administered the vehicle (Figure 4A), confirming the relevance of aerobic glycolysis to cancer progression in this model. However, the overall survival was reduced in the C26 tumor-bearing mice receiving heptelidic acid, while the same treatment did not impact survival of the control littermates (Figure 4B). Time-matched body composition analysis at the time when the C26 mice that were treated with heptelidic acid developed cachexia showed no effect of heptelidic acid in the control mice. In contrast, the C26 tumor-bearing mice that were treated with heptelidic acid exhibited significant splenomegaly and quadriceps muscle loss compared to their vehicle-treated counterparts, providing further evidence of an earlier onset of wasting and cachexia in heptelidic acid-treated mice compared to the vehicle-treated C26 mice (Figure 4C–E).

Comprehensive toxicity tests were performed in the C26 tumor-bearing and the control mice that were treated with heptelidic acid (Figure S3). No evidence of hepatic, pancreatic, or renal toxicity was detected. All the assessed electrolytes, glucose levels, and markers of metabolic function were unaffected by heptelidic acid administration. Changes in GAPDH activity in non-targeted organs of mice that were treated with heptelidic acid compared to their control counterparts were quantified (Figure S4). No major activity changes were observed in the kidney or pancreas. GAPDH activity was increased in the liver of the C26 untreated mice, most likely a secondary effect of the described neutrophil infiltration. A downregulation (0.5-fold decrease) of GAPDH activity in the liver of both the C26 and the control mice compared to the untreated controls was observed. Since toxicity screening showed no effects in liver function and all the experiments included all the required control groups, toxicity was not considered a confounding factor for the observed effects of heptelidic acid administration on tumor growth kinetics and the overall survival.

It might be expected that inhibiting glycolytic flux in neutrophils would result in immune cell down-modulation [29], but we observed a further increase in neutrophilia and splenomegaly in the pre-cachectic C26 mice following heptelidic acid administration. A possible explanation is that the inhibition of glycolysis is known to impair macrophage efferocytosis, the major mechanism of apoptotic neutrophil clearance in peripheral tissues [30].

While neutrophil counts of the control mice remained unchanged upon treatment, heptelidic acid prompted a further increase in the circulating neutrophilia and an increased neutrophilia in lung and liver in the pre-cachectic C26 mice, compared to vehicle-treated pre-cachectic C26 mice; the leukocyte counts were also elevated in these organs (Figure 4F–K). Our observations thus revealed a therapeutic paradox: systemic inhibition of aerobic glycolysis reduces tumor growth but accelerates cancer-associated cachexia, with a further increase in systemic neutrophilia and decreased survival. Thus, heptelidic acid treatment uncovers a correlation between survival and aerobic glycolysis in immune cells.

### 3.5. Short-Term Systemic Depletion of Neutrophils Impairs Glucose Homeostasis during Cancer Progression

To ascertain whether neutrophilia represents a generalized response to systemic metabolic stress, we studied immune changes in non-cancer-bearing and cancer-bearing mice subjected to 24 h total food restriction (TFR). Using this approach to model cachexia, we previously demonstrated that iatrogenic induction of caloric deficiency in the pre-cachectic stage is sufficient to reproduce the functional energetic deficiency that we term ‘metabolic stress’ observed during cachexia in mice and humans5.

Non-tumor-bearing mice showed an increase in both circulating and tissue-infiltrating neutrophils after TFR (Figure S5A–C), consistent with the upregulated expression of the neutrophil chemoattractant ligands CXCL2 and CXCL5, overexpression of the surface receptor CD36 that is implicated in several aspects that are related to fatty acid metabolism, and the downregulated gene expression of the regulator of lipid biosynthesis and adipogenesis sterol regulatory element-binding protein 1 (SREPB1c), and fatty acid synthase (FAS) in the livers of fasted mice (Figure S5D). These data suggest a generalized, cancer-independent response of neutrophil proliferation, mobilization, and infiltration to conditions of metabolic stress that is consistent with previous reports [31].

To assess the systemic consequences of neutrophil depletion, we injected mice with an anti-Gr1 depleting antibody: a rat IgG2b antibody that recognizes Ly6G and Ly6C surface markers and depletes neutrophils via the complement-mediated membrane attack complex. We used anti-Gr1 rather than the more neutrophil-specific anti-Ly6G antibody because anti-Ly6G has lower efficiency than anti-Gr1 with regard to depleting neutrophils [32,33,34].

Anti-Gr1 administration depleted neutrophils efficiently but displayed a short window of efficacy, with a compensatory rebound in the neutrophil levels after a few days of treatment, despite repeated administration every 48 h (Figure S5E,F). Therefore, studying the effect of long-term pharmacological neutrophil depletion on cancer progression and cachexia was not experimentally possible using this model. Anorexia represents a key pathologic event in clinical cachexia, thus we used this short-term depletion window to assess the contribution of neutrophils to the metabolic consequences of TFR.

**Figure 5:**
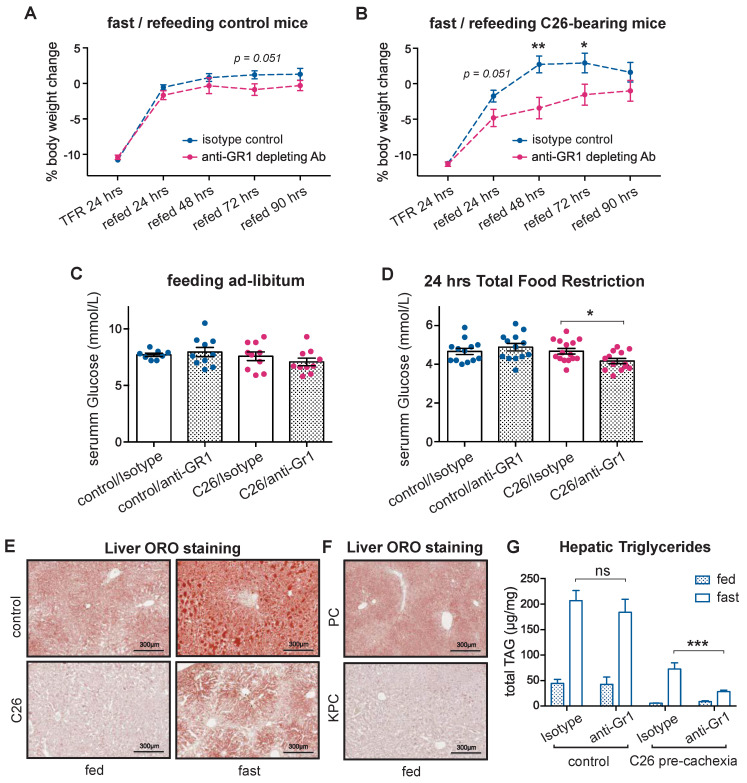
Effect of acute neutrophil depletion during cancer-associated metabolic stress. (**A**,**B**) Body weight trajectories after TFR of littermates (**A**) and pre-cachectic C26 mice (**B**) that were treated with anti-Gr1 or isotype. (**C**,**D**) The glucose levels in C26 mice and littermates that were treated with anti-Gr1 or isotype, when fed ad libitum (**C**) and after 24 h of total food restriction (TFR) (**D**); (**E**,**F**) Lipid staining by Oil Red O in the liver of fed or fasted pre-cachectic C26 mice and littermates (**E**) and the fed pre-cachectic KPC mice and the PC controls (**F**); (**G**) Quantification of triglycerides by liquid chromatography-mass spectrometry in the liver of fed and fasted pre-cachectic C26 mice and littermates that were treated with anti-Gr1 or isotype. The data are expressed as the mean ± SEM. Unpaired two-tailed Student’s *t*-tests were performed at each time point in (**A**,**B**), with the Holm–Šidák method correction for multiple comparisons. One-way ANOVA with Tukey’s correction for post hoc testing was used in (**C**,**D**,**G**). * *p*-value < 0.05, ** *p*-value < 0.01, *** *p*-value < 0.001, ns: not significant.

Non-tumor-bearing mice as well as pre-cachectic C26 tumor-bearing mice were treated with anti-Gr1 antibody or an isotype control and were challenged with TFR or allowed free access to food (Figure S5G). Body weight loss after TFR was similar in the control and the pre-cachectic C26 mice, and it was not affected by anti-Gr1 treatment (Figure S5H). However, the body weight recovery post-TFR was impaired in the anti-Gr1-treated C26 mice compared to the isotype-treated C26 mice and the controls, which fully recovered their original weight after 24 h of refeeding, suggesting dysfunctional macronutrient handling in the neutrophil-depleted, fasted C26 mice (Figure 5A,B). Although the serum glucose levels were similar in the non-tumor bearing controls and the pre-cachectic C26 mice in the fed state or after TFR (Figure 5C), neutrophil depletion caused hypoglycemia in the pre-cachectic C26 mice that were subjected to TFR (Figure 5D).

WAT lipolysis is essential for providing lipid substrates for hepatic gluconeogenesis, which sustains normal serum glucose levels during fasting [35]. Therefore, the hypoglycemic phenotype that was observed in the neutrophil-depleted pre-cachectic mice led us to investigate hepatic lipid metabolism. Lipid staining showed the expected physiological accumulation of hepatic lipids upon TFR in both controls and pre-cachectic C26 mice; however, the intensity was visibly reduced in the pre-cachectic C26 mice, both in the fed and TFR states (Figure 5E). Similarly, a decrease in hepatic lipid accumulation was observed in the livers from the pre-cachectic KPC mice compared to the control PC mice (Figure 5F). No obvious difference in hepatic glycogen staining was observed (Figure S5I). Quantification of the lipid triglycerides confirmed that the levels were reduced in livers from the pre-cachectic C26 mice, both in the TFR and the fed states. Remarkably, while neutrophil depletion did not affect the hepatic triglyceride levels in the non-tumor bearing controls, it significantly reduced the triglyceride levels in the fasted pre-cachectic C26 mice (Figure 5G). Collectively, these data support a model wherein neutrophil depletion reduces the availability of hepatic lipids, thereby impairing gluconeogenesis in cancer-bearing mice during conditions of acute metabolic stress, such as TFR. Thus, our observations indicate that cancer-associated neutrophilia and inflammation-induced neutrophil activation modulate substrate supply for systemic glucose homeostasis in cancer progression. These findings further support the notion that neutrophilia acts as an adaptive/protective mechanism that maintains systemic metabolic homeostasis during cancer progression.

## 4. Discussion

Cancer is a progressive disease that is initiated by cellular changes which promote tumor formation and often culminate in death of the organism. Overt cachexia represents an irreversible late stage of the disease and results from a sequence of metabolic events in cancer progression, including lean body mass wasting, hypercatabolism, weight loss, and insulin resistance. Since cancer-associated cachexia is a clear manifestation of the ongoing interactions between the tumor and the host metabolism, these interactions must occur throughout the stepwise process of cancer progression.

The findings we report here identify neutrophilia and aerobic glycolysis by neutrophils as an early display of tumor-host reciprocity that, when targeted both cellularly or metabolically, worsens the outcome and accelerates the cancer progression. This demonstrates an actively adaptive or protective response of the host against the advancing tumor that aims to sustain its metabolic homeostasis. Persisting efforts to achieve physiological balance may become depleting later on, and thereby trigger irreversible consequences in cancer progression, such as cancer-associated cachexia.

Regarding the translation of these findings, we note that neutrophilia in our in vivo models was first detectable in solid organs such as liver and lung, most likely due to active neutrophil recruitment, and only later in the circulating blood, which is the preferred medium of biomarker detection in the clinic. The later appearance of neutrophilia in the circulation may, therefore, falsely suggest a later onset of systemic dysregulation. In agreement with recent literature [36], we found that cancer-associated neutrophilia is mostly derived from increased splenic hematopoiesis, through expansion of HSC, and progenitors. Increased fractions of band cells or other neutrophil progenitors and/or metabolic reprogramming of the mature neutrophil population may contribute to the observed metabolic changes [37]. Mechanistically, splenic hematopoiesis is driven by local recruitment and accumulation of rare extramedullary HSC and progenitor cells in the splenic red pulp, mediated by the CCL2/CCR2 axis [38]. The spleen is known to contribute significantly to hematopoiesis in a range of inflammatory states [39,40,41], suggesting that this is an adaptive response to chronic inflammation. However, mice exhibit extramedullary hematopoiesis in the spleen more often than humans [42,43]. The factors driving neutrophilia in early disease remain unclear, and lack of relevant human samples at the right timepoints becomes an impediment to a direct translation of scientific findings. Yet, recent work suggests that primary tumor-driven ‘leaky gut syndrome’ can contribute to systemic inflammation. The expression of neutrophil chemokines in peripheral organs and the activated transcriptomic signatures of pre-cachectic mouse neutrophils further support the hypothesis that primary tumors trigger microbial-driven activation.

Such a putative protective role for neutrophils in cancer progression has diverse clinical implications because therapeutic agents that are given to patients with cancer frequently alter neutrophil counts. Chemotherapy, for example, can cause life-threatening neutropenia as a side effect, and blocking neutrophil migration via CXCR1 and CXCR2 [44], and inhibiting G-CSF [45], inhibit neutrophil function. Our model helps to partly explain the high mortality from neutropenic sepsis in patients with cancer [46], as infection and neutropenia would synergize to promote metabolic stress. On the other hand, steroids, G-CSF, and granulocyte-macrophage colony-stimulating factor (GM-CSF) increase neutrophil counts and our study suggests that this may have effects on systemic metabolic homeostasis.

A recent study showed that infiltration of neutrophils in the brain worsens cachexia severity [47] and future work should aim to delineate the contribution of organ-specific neutrophil infiltration. Thr expression of genes that are involved in NET formation and activation status in neutrophils from KPC mice is in agreement with the recent literature [48,49]. In this work, we focused on the definition of the systemic role of neutrophils in the pathophysiology of cachexia and used in vivo models of cancer that are associated with cachexia in an IL-6-dependent manner. Therefore, our conclusions are limited to specific cancer types. Studies using non-cachexia-inducing and IL-6-independent models of cancer would be required to generalize these findings and differentiate the effects that are caused by tumor progression from those that are driven by cancer-associated cachexia, as well as confirming that the protective role of neutrophils is a common mechanism. While the intrinsic cellular consequences of metabolic modulation have been thoroughly explored in neutrophils [8], their impact on host metabolism was unknown until now. We found that, in the C26 model of cancer that is associated with cachexia, neutrophils contribute to the hosts’ metabolic homeostasis during cancer progression and, thereby, suggest that treatments affecting neutrophils will have complex and potentially detrimental effects on the host. These results argue for the absolute necessity of a combined analysis regarding the effects of candidate cancer treatments on the tumor and the host.

Treatment effectiveness is often assessed using surrogate outcomes, such as a reduction in tumor size or increased time until radiological progression, termed “progression-free survival” [50], but it is increasingly recognized that these surrogate measures are poor predictors of overall survival [51]. Our results demonstrate that the host response is an important determinant of cancer outcome and may thus serve as an explanation of the insufficiency of tumor assessment to predict outcome. In this context, our finding that GAPDH inhibition leads to tumor regression but accelerated death is particularly striking. As heptelidic acid is expected to act only on tumor cells and immune cells, in particular activated neutrophils [23], this divergent phenotype indicates that the effect of GAPDH down-modulation on immune cells in the host outweighs its inhibitory effect on tumor growth. The targeting of upstream molecules, such as GLUT1, show similar effects in inhibiting tumor growth but may cause normal cell death because they prevent oxygen transport and endothelial cell angiogenesis for red blood cells [52,53]. These findings provide a proof-of-concept that paradoxical tumor-host responses to treatment occur in vivo, warranting further investigation in the context of other treatments [54].

## 5. Conclusions

Our findings highlight the importance of distinguishing adaptive from detrimental processes during cancer progression. The assumption that the reversal of a process that is observed during cancer progression will result in an improved outcome may not hold true. In fact, we demonstrate that targeting metabolic pathways that are relevant to both host and cancer cells may lead to a worsening of the outcome despite tumor regression.

## Data Availability

Publicly available datasets were analyzed in this study. This data can be downloaded from SRA (accession PRJNA749728).

## Supplementary Materials

The following are available online at https://www.mdpi.com/article/10.3390/cancers14040963/s1, Figure S1: Extended metabolic and immune phenotyping of C26 and KPC models of cancer cachexia, Figure S2: Extended characterization of the transcriptional profile of KPC-derived and PC-derived neutrophils, Figure S3: Toxicity screening of heptelidic acid treatment in the C26 and the control mice, Figure S4: Off-target GAPDH blockade of heptelidic acid treatment, Figure S5: Immune characterization of the C26 mouse model of cachexia, Figure S6: Raw immune cell counts in KPC and PC mice.

## References

[1] Li Z., Zhao R., Cui Y., Zhou Y., Wu X. (2018). The dynamic change of neutrophil to lymphocyte ratio can predict clinical outcome in stage I-III colon cancer. Sci. Rep..

[2] Dusselier M., Deluche E., Delacourt N., Ballouhey J., Egenod T., Melloni B., Vergnenegre C., Veillon R., Vergnenegre A. (2019). Neutrophil-to-lymphocyte ratio evolution is an independent predictor of early progression of second-line nivolumab-treated patients with advanced non-small-cell lung cancers. PLoS ONE.

[3] Luo J.L., Tan W., Ricono J.M., Korchynskyi O., Zhang M., Gonias S.L., Cheresh D.A., Karin M. (2007). Nuclear cytokine-activated IKKα controls prostate cancer metastasis by repressing Maspin. Nature.

[4] Kulbe H., Thompson R., Wilson J.L., Robinson S., Hagemann T., Fatah R., Gould D., Ayhan A., Balkwill F. (2007). The Inflammatory Cytokine Tumor Necrosis Factor-α Generates an Autocrine Tumor-Promoting Network in Epithelial Ovarian Cancer Cells. Cancer Res..

[5] Rius J., Guma M., Schachtrup C., Akassoglou K., Zinkernagel A.S., Nizet V., Johnson R.S., Haddad G.G., Karin M. (2008). NF-kappaB links innate immunity to the hypoxic response through transcriptional regulation of HIF-1alpha. Nature.

[6] Kortylewski M., Xin H., Kujawski M., Lee H., Liu Y., Harris T., Drake C., Pardoll D., Yu H. (2009). Regulation of the IL-23 and IL-12 balance by Stat3 signaling in the tumor microenvironment. Cancer Cell.

[7] Flint T.R., Janowitz T., Connell C.M., Roberts E.W., Denton A.E., Coll A.P., Jodrell D.I., Fearon D.T. (2016). Tumor-Induced IL-6 Reprograms Host Metabolism to Suppress Anti-tumor Immunity. Cell Metab..

[8] Petruzzelli M., Schweiger M., Schreiber R., Campos-Olivas R., Tsoli M., Allen J., Swarbrick M., Rose-John S., Rincon M., Robertson G. (2014). A switch from white to brown fat increases energy expenditure in cancer-associated cachexia. Cell Metab..

[9] Petruzzelli M., Wagner E.F. (2016). Mechanisms of metabolic dysfunction in cancer-associated cachexia. Genes Dev..

[10] Bindels L.B., Neyrinck A.M., Loumaye A., Catry E., Walgrave H., Cherbuy C., Leclercq S., Hul M., Plovier H., Pachikian B. (2018). Increased gut permeability in cancer cachexia: Mechanisms and clinical relevance. Oncotarget.

[11] Ghosh S.S., Wang J., Yannie P.J., Ghosh S. (2020). Intestinal Barrier Dysfunction, LPS Translocation, and Disease Development. J. Endocr. Soc..

[12] Fearon K.C.H., Glass D.J., Guttridge D.C. (2012). Cancer Cachexia: Mediators, Signaling, and Metabolic Pathways. Cell Metab..

[13] Temel J.S., Greer J.J., El-Jawahri A., Pirl W.F., Park E.R., Jackson V.A., Bacl A.L., Kamdar M., Jacobsen J., Chittenden E.H. (2017). Effects of Early Integrated Palliative Care in Patients with Lung and GI Cancer: A Randomized Clinical Trial. J. Clin. Oncol..

[14] Sadiku P., Willson J.A., Dickinson R.S., Murphy F., Harris A.J., Lewis A., Sammut D., Mirchandani A.S., Ryan E., Watts E.R. (2017). Prolyl hydroxylase 2 inactivation enhances glycogen storage and promotes excessive neutrophilic responses. J. Clin. Investig..

[15] Altenberg B., Greulich K.O. (2004). Genes of glycolysis are ubiquitously overexpressed in 24 cancer classes. Genomics.

[16] Mackey J.B.G., McFarlane A.J., Jamieson T., Jackstadt R., Raffo-Iraolagoitia X.L., Secklehner J., Cortes-Lavaus X., Fercoq F., Clarke Q., Hedley A. (2021). Maturation, developmental site, and pathology dictate murine neutrophil function. bioRxiv.

[17] Ewels P.A., Peltzer A., Fillinger S., Patel H., Alnberg J., Wilm A., Garcia M.U., Di Tommaso P., Nahnsen S. (2020). The nf-core framework for community-curated bioinformatics pipelines. Nat. Biotechnol..

[18] Howard R., Kanetsky P.A., Egan K.M. (2019). Exploring the prognostic value of the neutrophil-to-lymphocyte ratio in cancer. Sci. Rep..

[19] Barker T., Fulde G., Moulton B., Nadauld L.D., Rhodes T. (2020). An elevated neutrophil-to-lymphocyte ratio associates with weight loss and cachexia in cancer. Sci. Rep..

[20] Satake S., Hirai H., Hayashi Y., Shime N., Tamura A., Yao H., Yoshioka S., Miura Y., Inaba T., Fujita N. (2012). C/EBPβ Is Involved in the Amplification of Early Granulocyte Precursors during Candidemia-Induced “Emergency” Granulopoiesis. J. Immunol..

[21] Riffelmacher T., Clarke A., Richter F.C., Stranks A., Pandey S., Danielli S., Hublitz P., Yu Z., Johnson E., Schwerd T. (2017). Autophagy-Dependent Generation of Free Fatty Acids Is Critical for Normal Neutrophil Differentiation. Immunity.

[22] Kuhns D.B., Priel D.A.L., Chu J., Zarember K.A. (2015). Isolation and Functional Analysis of Human Neutrophils. Curr. Protoc. Immunol..

[23] Warburg O., Wind F., Negelein E. (1927). The metabolism of tumors in the body. J. Gen. Physiol..

[24] Jiang B. (2017). Aerobic glycolysis and high level of lactate in cancer metabolism and microenvironment. Genes Dis..

[25] Kornberg M.D., Bhargava P., Kim P.M., Putluri V., Snowman A.M., Putluri N., Calabresi P.A., Snyder S.H. (2018). Dimethyl fumarate targets GAPDH and aerobic glycolysis to modulate immunity. Science.

[26] Shestov A.A., Liu X., Ser Z., Cluntun A.A., Hung Y.P., Huang L., Kim D., Le A., Yellen G., Albeck J.G. (2014). Quantitative determinants of aerobic glycolysis identify flux through the enzyme GAPDH as a limiting step. eLife.

[27] Liberti M.V., Dai Z., Wardell S.E., Baccile J.A., Liu X., Gao X., Baldi R., Mehrmohamadi M., Johnson M.O., Madhukar N.S. (2018). Metabolic Control Analysis Uncovers a Predictive Model for Selective Targeting of the Warburg Effect through GAPDH Inhibition with a Natural Product. Cell Metab..

[28] Yun J., Mullarky E., Lu C., Bosch K.N., Kavalier A., Rivera K., Roper J., Chio I.I., Giannopoulou E.G., Rago C. (2015). Vitamin C selectively kills KRAS and BRAF mutant colorectal cancer cells by targeting GAPDH. Science.

[29] Veiga-da-Cunha M., Chevalier N., Stephenne X., Defour J.P., Paczia N., Ferster A., Achouri Y., Dewulf J.P., Linster C.L., Bommer G.T. (2019). Failure to eliminate a phosphorylated glucose analog leads to neutropenia in patients with G6PT and G6PC3 deficiency. Proc. Natl. Acad. Sci. USA.

[30] Morioka S., Perry J.S.A., Raymond M.H., Medina C.B., Zhu Y., Zhao L., Serbulea V., Onengut-Gumuscu S., Leitinger N., Kucenas S. (2018). Efferocytosis induces a novel SLC program to promote glucose uptake and lactate release. Nature.

[31] Talukdar S., Oh D.Y., Bandyopadhyay G., Li D., Xu J., McNelis J., Lu M., Li P., Yan Q., Zhu Y. (2012). Neutrophils mediate insulin resistance in high fat diet fed mice via secreted elastase. Nat. Med..

[32] Boivin G., Faget J., Ancey P.B., Gkasti A., Mussard J., Engblom C., Pfirschke C., Contat C., Pascual J., Vazquez J. (2020). Durable and controlled depletion of neutrophils in mice. Nat. Commun..

[33] Wojtasiak M., Pickett D.L., Tate M.D., Londrigan S.L., Bedoui S., Brooks A.G., Reading P.C. (2010). Depletion of Gr-1+, but not Ly6G+, immune cells exacerbates virus replication and disease in an intranasal model of herpes simplex virus type 1 infection. J. Gen. Virol..

[34] Faget J., Boivin G., Ancey P.B., Gkasti A., Mussard J., Engblom C., Pfirschke C., Vazquez J., Bondriss-Vermare N., Caux C. (2018). Efficient and specific Ly6G + cell depletion: A change in the current practices toward more relevant functional analyses of neutrophils. bioRxiv.

[35] Petersen M.C., Shulman G.I. (2018). Mechanisms of Insulin Action and Insulin Resistance. Physiol. Rev..

[36] Cortez-Retamozo V., Etzrodt M., Newton A., Rauch P.J., Chudnovsky A., Berger C., Ryan R.J.H., Iwamoto Y., Marinelli B., Gorbatov R. (2012). Origins of tumor-associated macrophages and neutrophils. Proc. Natl. Acad. Sci. USA.

[37] Rice M.C., Davies C.L., Subleski J.J., Maio N., Gonzalez-Cotto M., Andrews C., Patel N.K., Palmieri E.M., Weiss J.M., Lee J. (2018). Tumour-elicited neutrophils engage mitochondrial metabolism to circumvent nutrient limitations and maintain immune suppression. Nat. Commun..

[38] Wu C., Huiheng N., Mingyu L., Jie L., Shufeng L., Wenjie Z., Jing X., Wen-Chao W., Jing L., Chun-Kui S. (2018). Spleen mediates a distinct hematopoietic progenitor response supporting tumor-promoting myelopoiesis. J. Clin. Investig..

[39] Griseri T., McKenzie B.S., Schiering C., Powrie F. (2012). Dysregulated Hematopoietic Stem and Progenitor Cell Activity Promotes Interleukin-23-Driven Chronic Intestinal Inflammation. Immunity.

[40] Burberry A., Zeng M.Y., Ding L., Wicks I., Inohara N., Morrison S.J., Nunez G. (2014). Infection Mobilizes Hematopoietic Stem Cells through Cooperative NOD-like Receptor and Toll-like Receptor Signaling. Cell Host Microbe.

[41] Regan-Komito D., Swann J.W., Demetriou P., Cohen E.S., Horwood N.J., Sansom S.N., Griseri T. (2020). GM-CSF drives dysregulated hematopoietic stem cell activity and pathogenic extramedullary myelopoiesis in experimental spondyloarthritis. Nat. Commun..

[42] Bozzini C.E., Rendo M.E.B., Devoto F.C., Epper C.E. (1970). Studies on medullary and extramedullary erythropoiesis in the adult mouse. Am. J. Physiol. Content.

[43] Schubert T.E.O., Obermaier F., Ugocsap P., Mannel D.N., Echtenacher B., Hofstadter F., Haerle P. (2008). Murine Models of Anaemia of Inflammation: Extramedullary Haematopoiesis Represents a Species Specific Difference to Human Anaemia of Inflammation That Can Be Eliminated by Splenectomy. Int. J. Immunopathol. Pharmacol..

[44] Bertini R., Allegreti M., Bizzarri C., Moriconi A., Locati M., Zampella G., Cervellera M.N., Di Cioccio V., Cesta M.C., Galliera E. (2004). Noncompetitive allosteric inhibitors of the inflammatory chemokine receptors CXCR1 and CXCR2: Prevention of reperfusion injury. Proc. Natl. Acad. Sci. USA.

[45] Ries C.H., Cannarile M.A., Hoves S., Benz J., Wartha K., Runza V., Rey-Giraud F., Pradel L.P., Feuerhake F., Klaman I. (2014). Targeting Tumor-Associated Macrophages with Anti-CSF-1R Antibody Reveals a Strategy for Cancer Therapy. Cancer Cell.

[46] Legrand M., Max A., Peigne V., Mariotte E., Canet E., Debrumetz A., Lemiale V., Seguin A., Darmon M., Schlemmer B. (2012). Survival in neutropenic patients with severe sepsis or septic shock. Crit. Care Med..

[47] Burfeind K.G., Zhu X., Norgard M.A., Levasseur P.R., Huisman C., Buenafe A.C., Olson B., Michaelis K.A., Torres E.R.S., Jeng S. (2020). Circulating myeloid cells invade the central nervous system to mediate cachexia during pancreatic cancer. eLife.

[48] Munir H., Jones J.O., Janowitz T., Hoffman M., Euler M., Martins C.P., Welsh S.J., Shields J.D. (2021). Stromal-driven and Amyloid β-dependent induction of neutrophil extracellular traps modulates tumor growth. Nat. Commun..

[49] Schmid M.C., Khan S.Q., Kaneda M.M., Pathria P., Shepard R., Louis T.L., Anand S., Woo G., Leem C., Faridi M.H. (2018). Integrin CD11b activation drives anti-tumor innate immunity. Nat. Commun..

[50] Michaelis L.C., Ratain M.J. (2006). Measuring response in a post-RECIST world: From black and white to shades of grey. Nat. Cancer.

[51] Prasad V., Kim C., Burotto M., Vandross A. (2015). The Strength of Association between Surrogate End Points and Survival in Oncology. JAMA Intern. Med..

[52] Wu Q., Ba-alawi W., Deblois G., Cruickshank J., Duan S., Lima-Fernandes E., Haight J., Tonekaboni S.A.M., Fortier A.M., Kuasne H. (2020). GLUT1 inhibition blocks growth of RB1-positive triple negative breast cancer. Nat. Commun..

[53] Meng Y., Xu X., Luan H., Li L., Dai W., Li Z., Bian J. (2019). The progress and development of GLUT1 inhibitors targeting cancer energy metabolism. Future Med. Chem..

[54] Janowitz T. (2018). Cancer: The Tumor-Driven Disease of the Host. Cell Metab..

