# Supplementary material for: Early Neutrophilia Marked by Aerobic Glycolysis Sustains Host Metabolism and Delays Cancer Cachexia
Source: Cancers (Basel). 2022 Feb 15;14(4):963. doi: 10.3390/cancers14040963 (PMC8870098; doi:10.3390/cancers14040963)
Supplement: Supplementary file 1 [file cancers-14-00963-s001.zip › cancers-1462264-supplementary.pdf]

cancers

MDPI

Supplementary Materials: Early Neutrophilia Marked by Aerobic Glycolysis Sustains Host Metabolism and Delays Cancer Cachexia

Michele Petruzzelli, Miriam Ferrer, Martijn J. Schuijs, Sam O. Kleeman, Nicholas Mourikis, Zoe Hall, David Perera, Shwethaa Raghunathan, Michele Vacca, Edoardo Gaude, Michael J. Lukey, Duncan I. Jodrell, Christian Frezza, Erwin F. Wagner, Ashok R. Venkitaraman, Timotheus Y. F. Halim and Tobias Janowitz

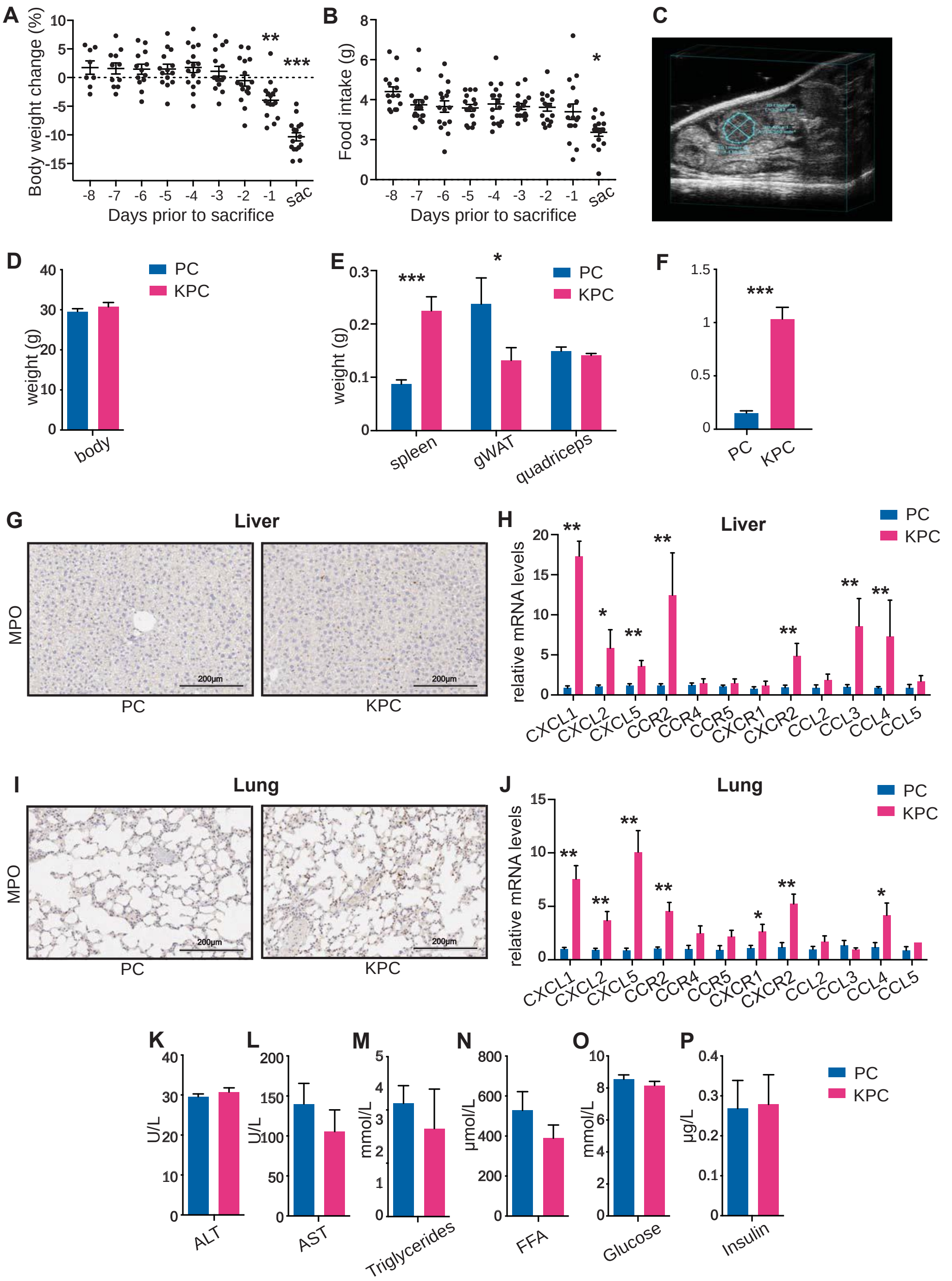

Figure S1. Extended metabolic and immune phenotyping of C26 and KPC models of cancer cachexia. (**A**,**B**) Longitudinal change in % body weight (**A**) and food intake changes (**B**) during the last

8 days prior to sacrifice in mice injected subcutaneously with C26 colorectal cancer cells; (**C**) Representative ultrasound image of a pancreatic tumor in a pre-cachectic KPC mouse; (**D**,**E**) Body (**D**), spleen, gonadal white adipose tissue (gWAT) and quadricep (**E**) weights of pre-cachectic KPC mice and PC littermates; (**F**) Neutrophil to lymphocyte ratio (NLR) in pre-cachectic KPC mice and PC controls; (**G**,**I**) Myeloperoxidase (MPO) staining as marker for neutrophil infiltration in the liver (**G**) and lung (**I**) of pre-cachectic KPC mice and PC controls, and relative expression levels of chemokines and chemokine receptors measured by RT-qPCR in the liver (**H**) and lung (**J**) of pre-cachectic KPC and control PC mice; (**K**–**P**) Quantification of liver transaminases (**K**,**L**), triglycerides (**M**), free fatty acids (FFA) (**N**), glucose (**O**), and insulin (**P**) in the plasma of pre-cachectic KPC mice and PC controls. Data are expressed as the mean ± SEM. In (**A**,**B**) statistical differences were examined using multiple t-tests (one per each time point) and the Holm-Sidak method to correct for multiplicity, with alpha = 0.05. In (**D**–**P**) unpaired two-tailed Student's t-test with Welch's correction was applied. * p-value < 0.05, ** p-value < 0.01, *** p-value < 0.001.

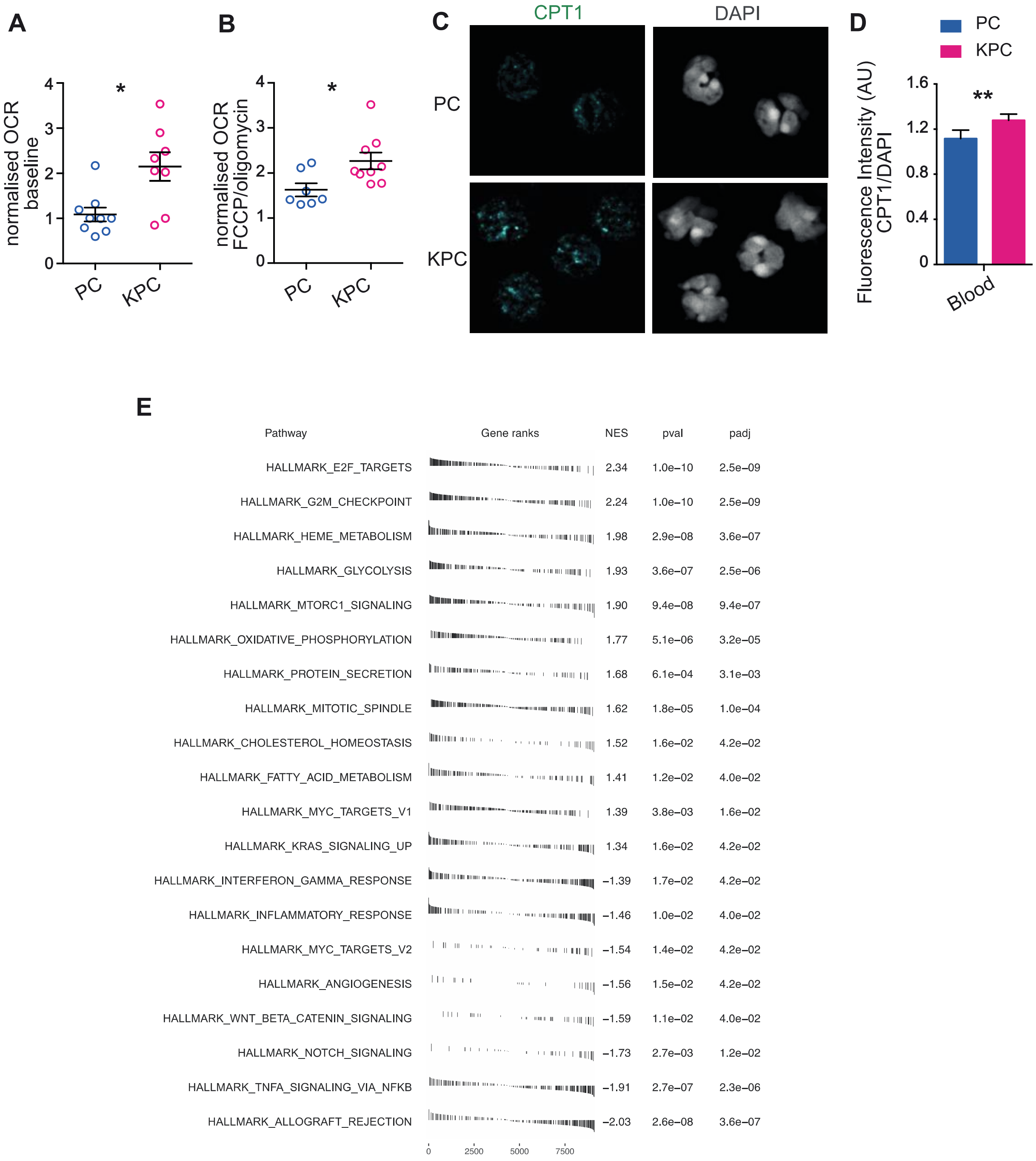

Figure S2. Extended characterization of the transcriptional profile of KPC-derived and PC-derived neutrophils. (**A**,**B**) Normalized oxygen consumption rate (OCR) measurements by Seahorse assay at baseline (**A**) and after treatment with phenylhydrazone (FCCP) and oligomycin (**B**) in circulating leukocytes from pre-cachectic KPC mice and PC controls; (**C**) Representative immunofluorescence staining for carnitine palmitoyltransferase 1 (CPT1) and DAPI in sorted circulating neutrophils; (**D**) Quantification of the fluorescence intensity (AU) of CPT1 staining relative to DAPI in sorted circulating neutrophils. (**E**) Differential expression analysis of genes in isolated neutrophils of KPC compared to wild-type C57BL/6J mice. Data in (**A**,**B**,**D**) are expressed as the mean ± SEM. Unpaired two-tailed Student's t-test with Welch's correction was applied. * p-value < 0.05, ** p-value < 0.01, Statistical analysis performed in (**E**) is detailed in the Methods section.

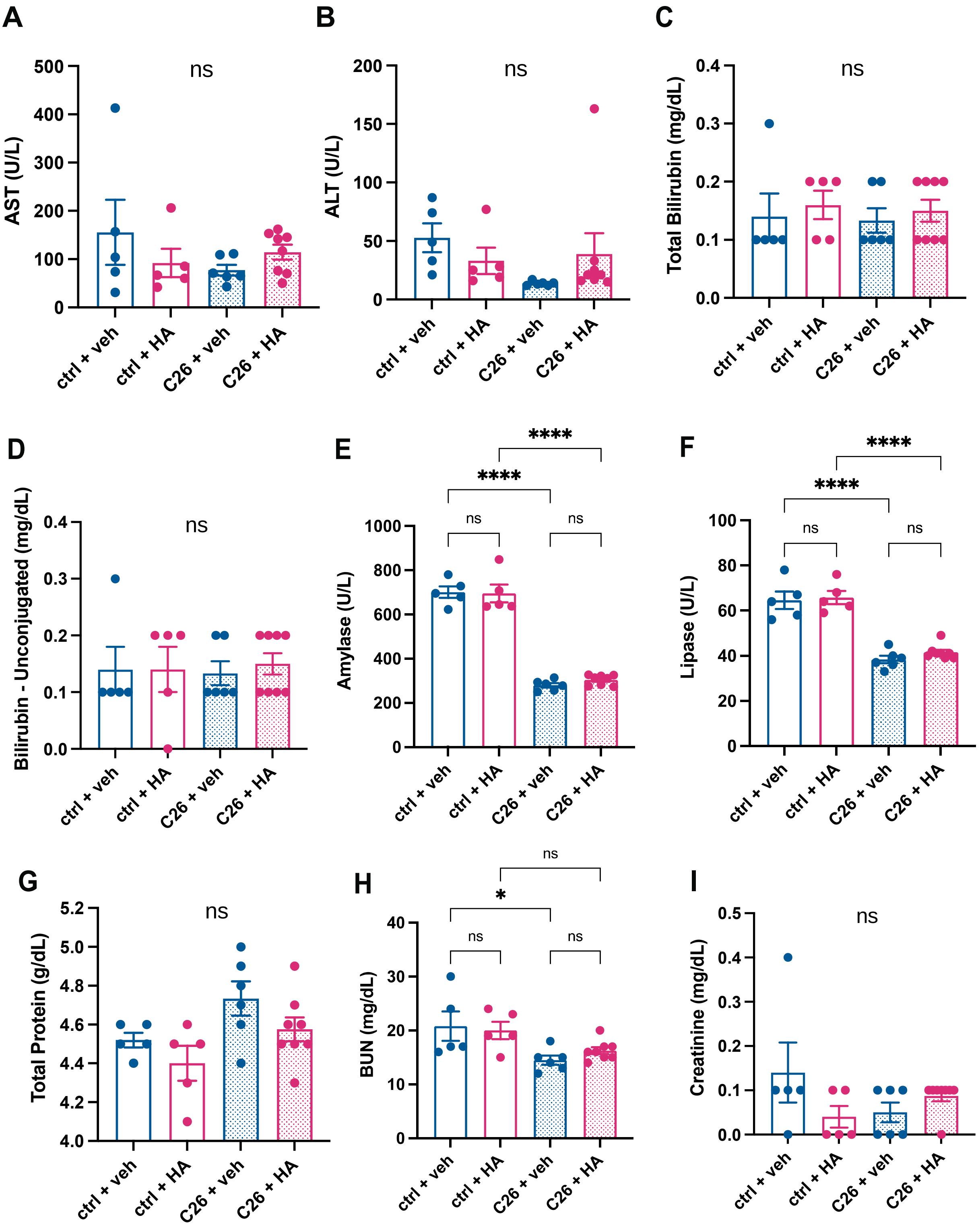
A
AST (U/L)
ns
B
ALT (U/L)
ns
C
Total Bilirubin (mg/dL)
ns
D
Bilirubin - Unconjugated (mg/dL)
ns
E
Amylase (U/L)
****
****
ns
ns
F
Lipase (U/L)
****
****
ns
ns
G
Total Protein (g/dL)
ns
H
BUN (mg/dL)
ns
*
ns
ns
I
Creatinine (mg/dL)
ns
ctrl + veh
ctrl + HA
C26 + veh
C26 + HA

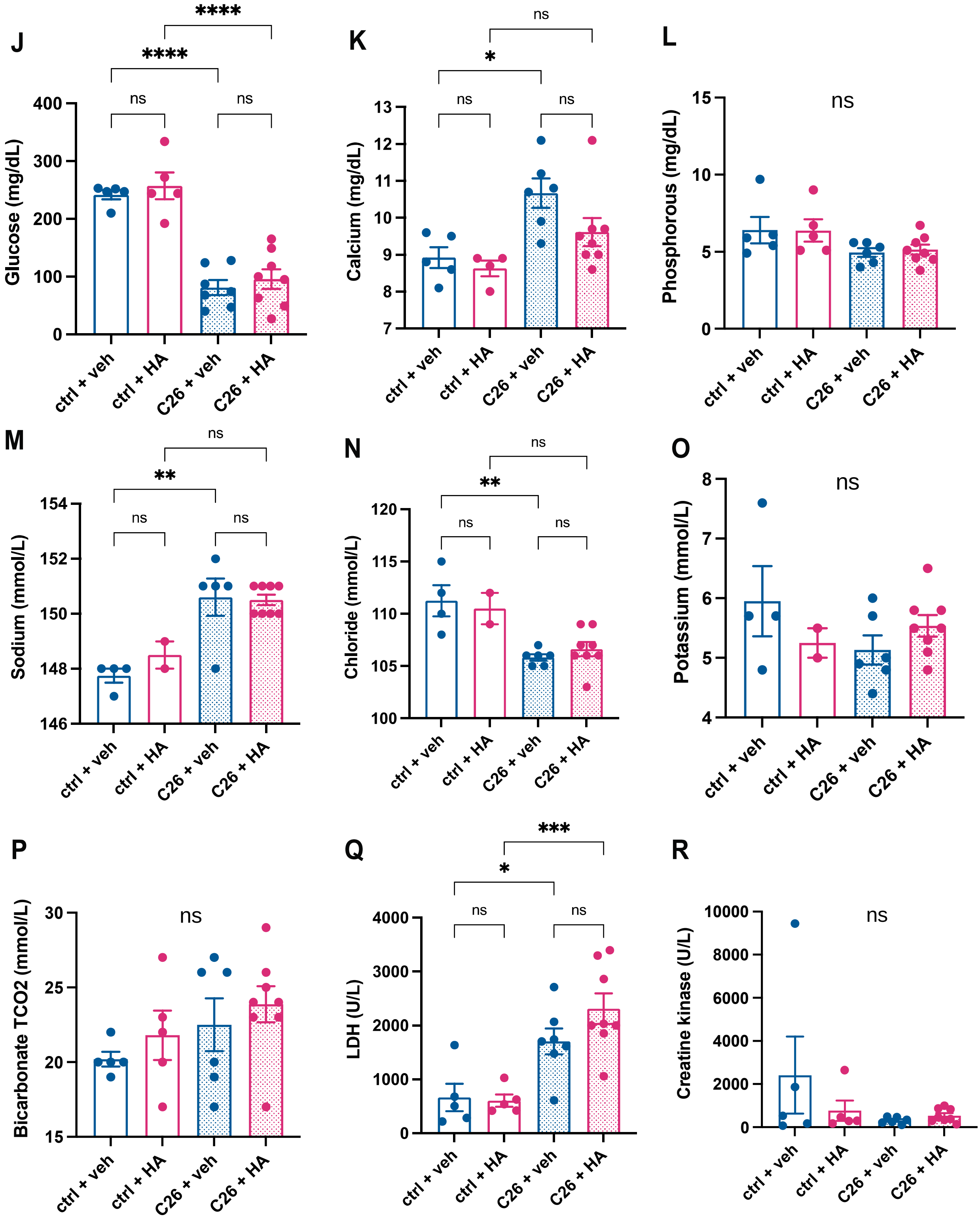
J
Glucose (mg/dL)
****
****
ns
ns
ctrl + veh
ctrl + HA
C26 + veh
C26 + HA
K
Calcium (mg/dL)
ns
*
ns
ns
L
Phosphorous (mg/dL)
ns
M
Sodium (mmol/L)
ns
**
ns
ns
N
Chloride (mmol/L)
ns
**
ns
ns
O
Potassium (mmol/L)
ns
P
Bicarbonate TCO2 (mmol/L)
ns
Q
LDH (U/L)
***
*
ns
ns
R
Creatine kinase (U/L)
ns

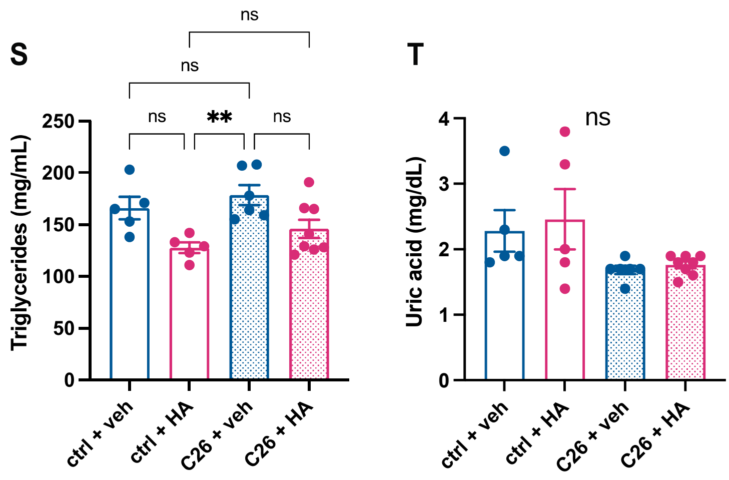

Figure S3. Toxicity screening of heptelidic acid treatment in C26 and control mice. (**A**,**B**) Quantification of the liver transaminases (**A**) Aspartate aminotransferase (AST) and (B) Alanine aminotransferase (ALT); (**C**,**D**) Bilirubin measurements (**C**) total, and (**D**) unconjugated; (**E**,**F**) Blood levels of the pancreatic enzymes (**E**) Amylase, and (**F**) Lipase; (**G**) Quantification of protein in the blood; (**H**,**I**) Kidney function tests of (**H**) Blood urea nitrogen (BUN), and (**I**) creatinine; (**J**) Cirulating glucose levels; (**K**–**O**) Measuremtens of circulating minerals and electrolytes (**K**) Calcium, (**L**) Phosphorous, (M) Sodium, (N) Chloride, and (**O**) Potassium; (**P**) Total CO2 levels in the blood; (**Q**) Lactate hydrogenase (LDH) quantification; (**R**) Levels of creatine kinase to asses musle damage; (**S**) Circulating triglyrecide levels, and (**T**) uric acid. Data are expressed as the mean ± SEM. One-way ANOVA with Tukey's correction for post-hoc testing was used for statistical comparison. * *p*-value < 0.05, ** *p*-value < 0.01, *** *p*-value < 0.001, **** *p*-value < 0.0001, ns: not significant.

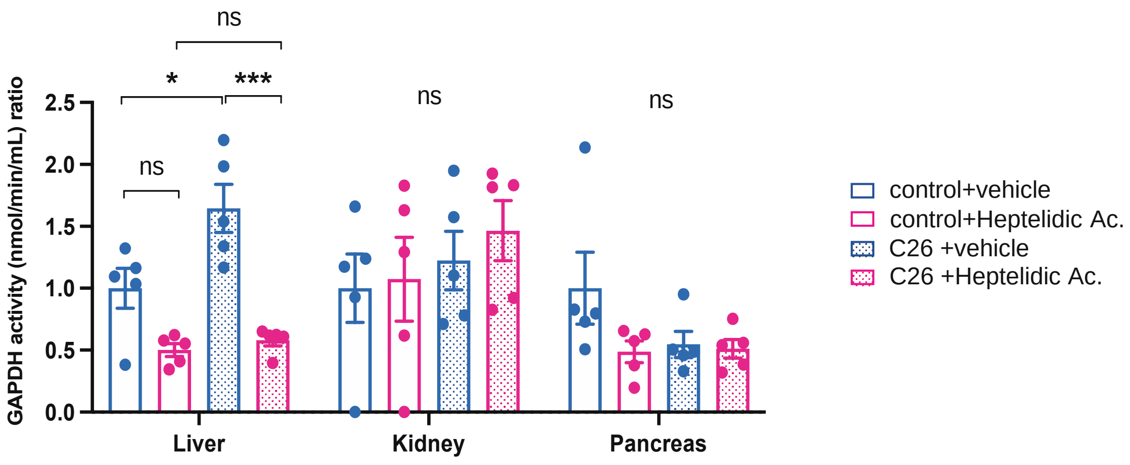

Figure S4. Off-target GAPDH blockade of heptelidic acid treatment. Data are expressed as the mean ± SEM. One-way ANOVA with Tukey's correction for post-hoc testing was used for statistical comparison. * *p*-value < 0.05, *** *p*-value < 0.001, ns: not significant.

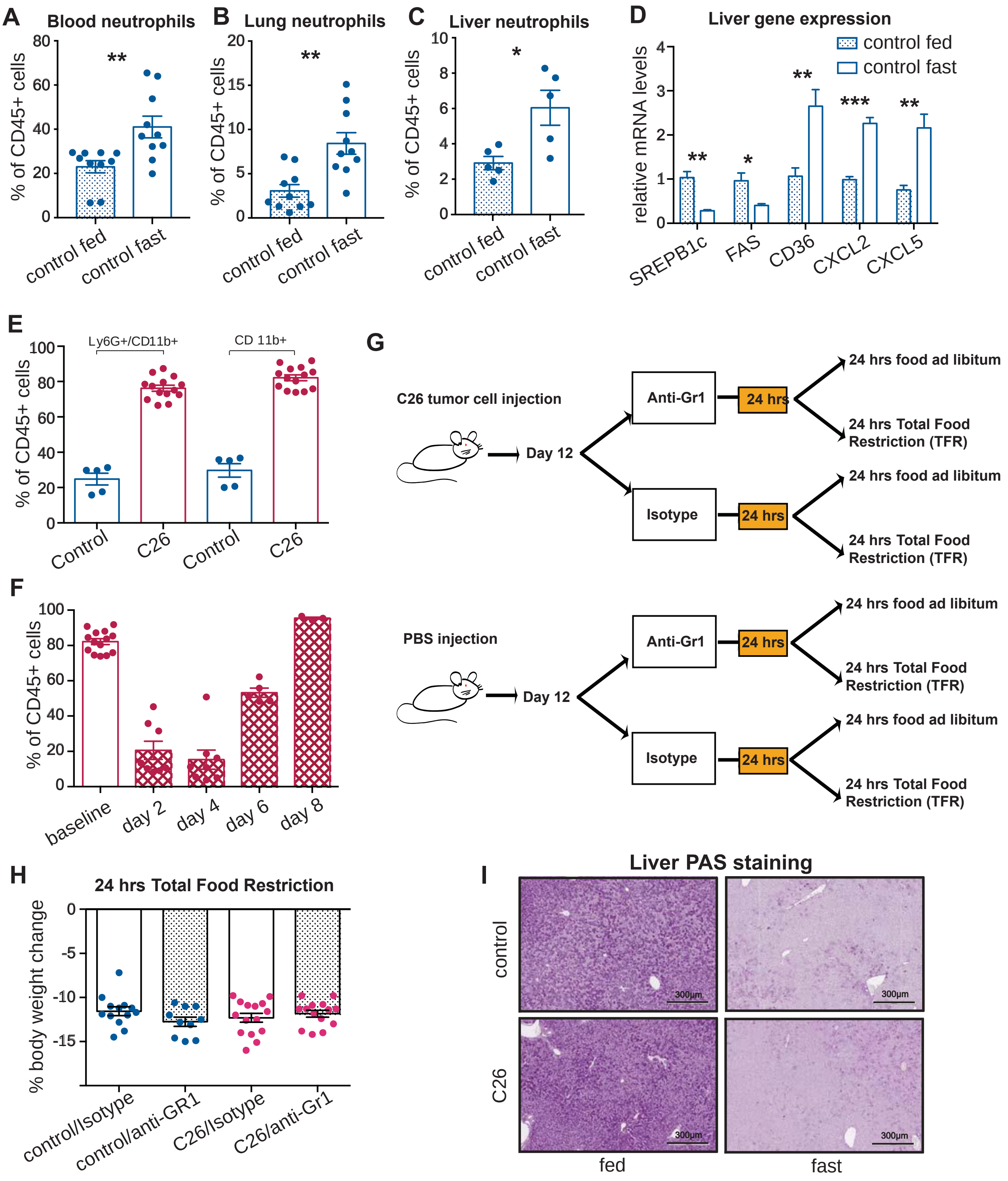

Figure S5. Immune characterization of the C26 mouse model of cachexia. (**A**–**C**) Quantification of neutrophils (displayed as % of all CD45+ cells) by flow cytometry in the blood (**A**), lung (**B**), and liver (**C**) of control mice fed and after 24 hours of total food restriction (TFR); (**D**) Gene expression levels of metabolic genes and chemoattractant ligands in the liver of mice in Fig EV3C-E; (**E**) Quantification by flow cytometry of Ly6G+/CD11b+ and CD11b+ cells (displayed as % out of all CD45+ cells) in the blood of C26 mice and littermates; (**F**) Quantification by flow cytometry of CD11b+ (displayed as % out of all CD45+ cells) in the blood of C26 mice at baseline and every other day after starting treatment with anti-Gr1; (**G**) Schematic representation of the TFR and neutrophil depletion experiments; (**H**) Percentage body weight changes after TFR in C26 mice and controls treated with anti-Gr1 or isotype; (**I**) Periodic acid-Schiff (PAS) staining of glycogen in the liver of fed or fasted pre-cachectic C26 mice and controls. Data are expressed as the mean ± SEM. In (A-D) statistical

differences were examined using unpaired two-tailed Student's t-test with Welch's correction. One-way ANOVA with Tukey's correction for post-hoc testing was used in (H). * *p*-value < 0.05, ** *p*-value < 0.01.

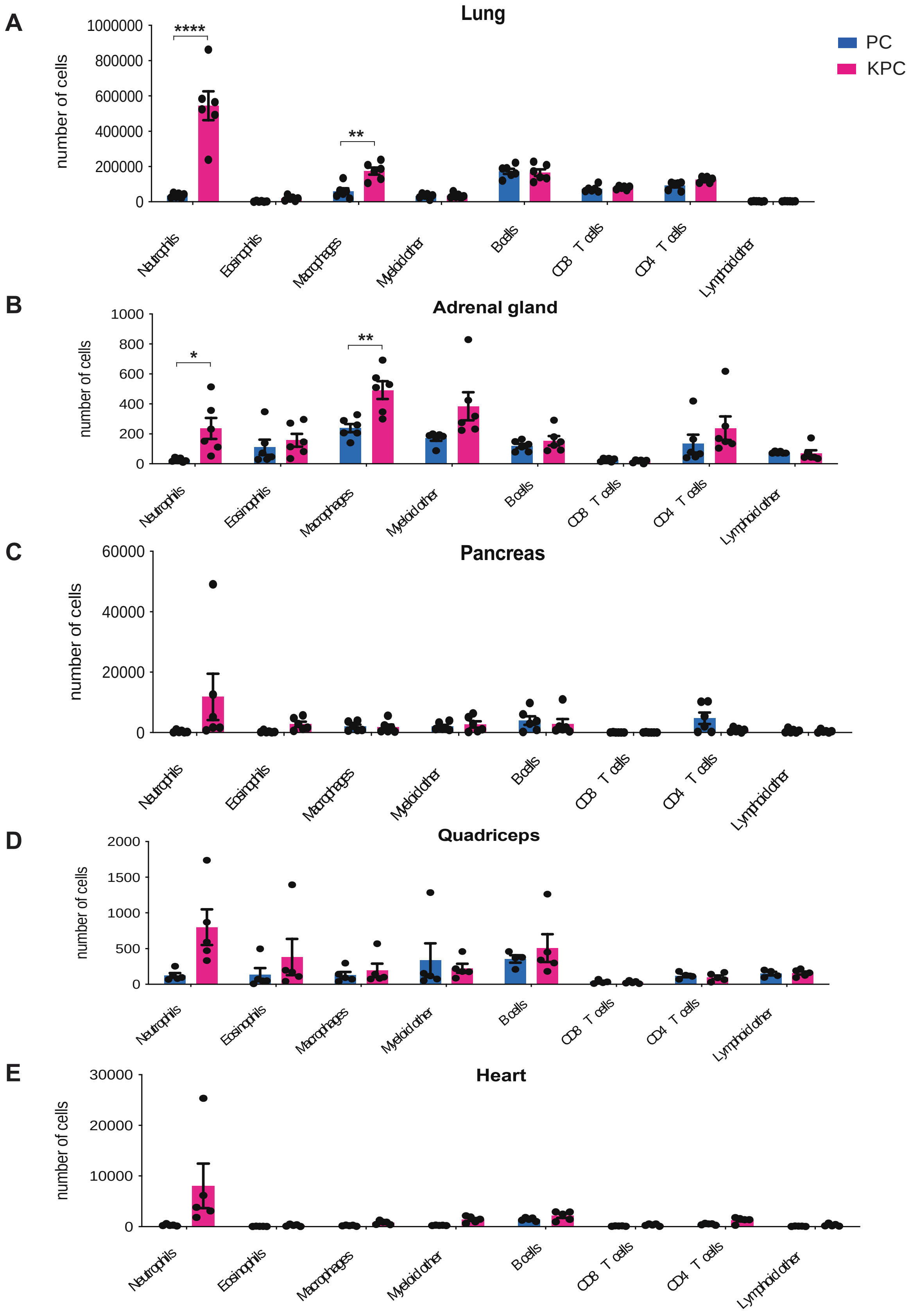

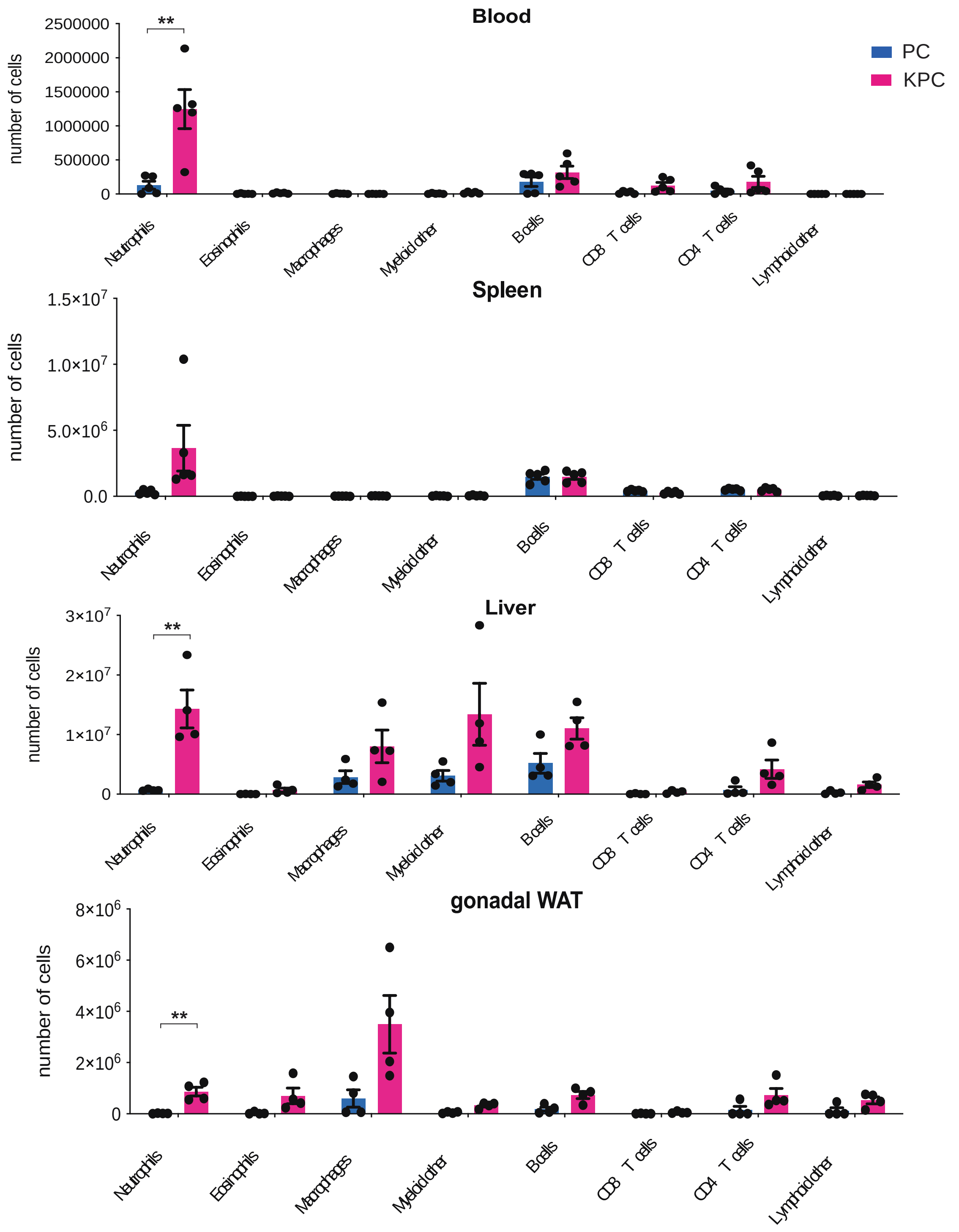

Figure S6. Raw immune cell counts in KPC and PC mice. (**A**–**I**) Raw quantification of immune cells (displayed as number of cells) in lung (**A**), adrenal gland (**B**), pancreas (**C**), quadriceps (**D**), heart (**E**), blood (**F**), spleen(**G**), liver (**H**), and gonadal WAT (**I**). Data are expressed as the mean ± SEM. Unpaired two-tailed Student's t-tests with Welch's correction were applied. * p-value < 0.05, ** p-value < 0.01, **** p-value < 0.0001.
